# An Update on Clinically Advanced PROTAC Degraders and Their Synthesis

**DOI:** 10.3390/molecules31010033

**Published:** 2025-12-22

**Authors:** Ranjan Kumar Acharyya, Yugandhar Kothapalli, Suresh Yarlagadda, Chayan K. De, Srinivasa Rao Allu, Joyeeta Roy, Rohan Kalyan Rej

**Affiliations:** 1 Rogel Cancer Center, Department of Internal Medicine, University of Michigan, Ann Arbor, MI 48109, USA; 2 Department of Pharmaceutical and Biomedical Sciences, College of Pharmacy, University of Georgia, Athens, GA 30602, USA; 3 Department of Chemistry, Purdue University, 720 Clinic Drive, West Lafayette, IN 47907, USA; 4 Department of Urology, University of Michigan, Ann Arbor, MI 48109, USA; 5 Department of Medicinal Chemistry, College of Pharmacy, University of Michigan, Ann Arbor, MI 48109, USA

**Keywords:** proteolysis-targeting chimeras (PROTACs), target protein degradation (TPD), cancer, anti-cancer agents

## Abstract

Proteolysis-targeting chimeras (PROTACs) have emerged as a revolutionary therapeutic modality that enables degradation of therapeutically relevant proteins through the protein disposal machinery, the ubiquitin-proteasome system (UPS). Unlike traditional small-molecule inhibitors, PROTACs harness bifunctional molecules to induce targeted protein degradation, offering advantages such as increased specificity, catalytic activity, and the potential to address previously undruggable targets. Since their conception 20 years ago, PROTACs have made significant strides in target protein degradation (TPD), and today, PROTACs are on the verge of their first clinical approval. This review presents a detailed overview of PROTAC targets, clinical development progress, and the design and detailed synthesis of degrader molecules that have advanced to clinical trials.

## 1. Introduction

Targeted protein degradation has emerged as a transformative concept in modern drug discovery, offering novel strategies for intervention in cellular processes previously deemed “undruggable.” In 2001, Sakamoto et al. revolutionized the field of protein degradation with the first demonstration of the concept of PROTAC technology [1,2,3]. Over the next decade, this innovative approach captured the attention of biotech companies and academic institutions. Among the pioneering advancements were Bavdegalutamide (ARV-110) and Vepdegestrant (ARV-471), which were among the first compounds to enter clinical trials [4,5]. On 8 August 2025 FDA has accepted a New Drug Application (NDA) for Vepdegestrant for the treatment of ESR1-mutated ER+/HER2-advanced breast cancer patients based on the pivotal Phase 3 VERITAC-2 clinical trial (NCT05654623), demonstrating statistically significant and clinically meaningful improvement in median progression-free survival (PFS) compared to Fulvestrant [6,7].

**Figure 1:**
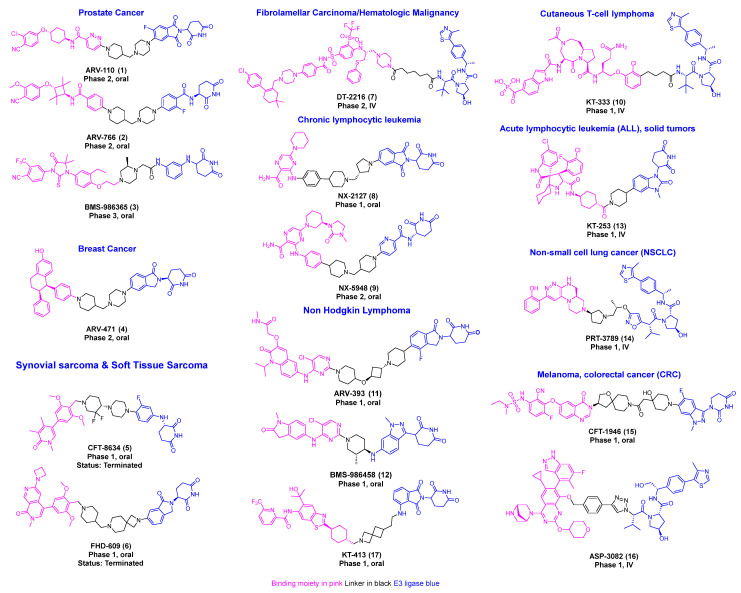
Disclosed structures of PROTACs in the clinical trial.

Recent research indicates a significant rise in the utilization of different E3 ligases in PROTAC development, highlighting the expanding scope of targeted protein degradation strategies [8,9,10]. Among these, E3 ligases Von Hippel–Lindau (VHL) and Cereblon (CRBN)-based PROTAC degraders have progressed to clinical development [11,12,13,14]. PROTAC technology has now been successfully used to target more than 100 proteins. This review offers a comprehensive overview of disclosed PROTAC degrader cancer targets, clinical development progress, and the design and detailed synthesis of degrader molecules (Figure 1) [13,15].

## 2. Discussion of Targets, Clinical Development Progress, and the Design and Detailed Synthesis of Degrader Molecules

### 2.1. PROTACS Targeting Androgen Receptor (AR)

Prostate cancer is the second most common cancer in men, causing over 400,000 deaths annually, with new cases expected to rise from 1.4 million in 2020 to 2.9 million by 2040 [16,17,18]. The androgen receptor (AR), a nuclear hormone receptor, plays a central role in the biology of prostate cancer. The AR is a 110 kDa transcription factor composed of an *N*-terminal activation function-1 (AF1) domain, a DNA-binding domain, a hinge region, and a ligand-binding domain (LBD, also known as AF2). In its inactive state, AR exists as a monomer bound to heat shock proteins (HSPs). Upon binding dihydrotestosterone (DHT), the HSPs dissociate, allowing AR to dimerize, translocate to the nucleus, and bind androgen response elements (AREs), thereby activating the transcription of genes that promote tumor growth and survival [19,20].

Current treatment approaches include surgical removal of the prostate and androgen deprivation therapy (ADT). While antiandrogen therapies are initially effective in clinical settings, AR mutations frequently lead to resistance. These mutations can convert AR antagonists into partial agonists and increase the receptor’s sensitivity to other steroid hormones such as estradiol, progesterone, and hydrocortisone. This process contributes to the development of lethal castration-resistant prostate cancer (CRPC), which remains incurable to date [21].

Clinical Development and Outcomes of Bavdegalutamide (ARV-110), Luxdegalutamide (ARV-766) and Gridegalutamide (BMS-986365): AR degraders present a promising strategy to overcome acquired AR mutations, as they enable the elimination of the receptor regardless of whether a mutation shifts a ligand’s effect from antagonism to agonism. ARV-110 (Bavdegalutamide), developed by Arvinas, was the first PROTAC-based AR degrader to enter clinical trials in 2019 for mCRPC (NCT03888612). However, Phase II results, measured by the prostate-specific antigen 50% reduction (PSA50), revealed that its efficacy was confined mainly to a small subgroup of patients with AR T878A/S or H875Y mutations. Among 140 patients with biomarker and PSA follow-up, those with AR T878A/S and/or H875Y mutations showed PSA declines of 46% (PSA50) and 58% (PSA30), compared to 10% and 23%, respectively, in patients without these mutations [4,22]. In a group of 7 patients with these mutations assessed by RECIST (Response Evaluation Criteria in Solid Tumors) criteria, 6 showed tumor shrinkage, including two confirmed partial responses. Of those with only one prior novel hormonal agent (NHA) and no chemotherapy, 5 out of 19 patients (26%) achieved PSA50. Overall, response rates were 16% for PSA50 and 29% for PSA30 [23].

**Figure 2:**
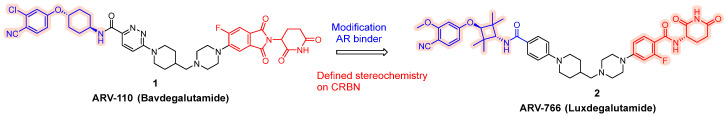
Discoveries along the way to ARV-766 (2).

To expand its AR-targeting portfolio and treat a broader range of prostate cancer patients, Arvinas developed Luxdegalutamide (ARV-766), a second-generation AR degrader designed for enhanced potency and efficacy. The discovery of ARV-766 was presented at AACR 2023. Key structural optimizations leading to Luxdegalutamide included replacing the six-membered ring in the POI moiety of ARV-110 with a tetramethyl cyclobutane ring, substituting a chlorine atom with a methoxy group in the AR binder, and modifying the CRBN component with precise stereochemistry (**ARV-766**, **2**, Figure 2).

ARV-766 has demonstrated robust degradation activity against multiple clinically relevant AR mutants, including wild-type AR as well as L702H (which occurs in up to 24% of treated mCRPC patients), W742C, T878A/D891H, S889G, M750V, T878A/S889G, and the dual mutant F877L/T878A. This broad mutational coverage supports its potPential for clinical benefit across a broader patient population. Importantly, ARV-766 does not degrade key IMiD neosubstrates such as GSPT1, Aiolos, Ikaros, CK1α, and SALL4; an attribute that may translate into a more favorable safety profile [24].

As of 15 December 2023, a total of 103 patients had been treated with ARV-766 (34 in Phase 1 and 69 in Phase 2). Among 28 PSA-evaluable patients with AR LBD mutations, the PSA50 response rate was 50.0% [24]. Given the increasing prevalence of the L702H mutation, more patients stand to benefit from the robust efficacy profile that ARV-766 offers. Furthermore, ARV-766 demonstrated a significantly improved tolerability profile compared to ARV-110, with markedly lower rates of fatigue, nausea, vomiting, and diarrhea. In conjunction with its compelling efficacy data, this favorable safety profile positions ARV-766 as a “superior” PROTAC AR degrader for both mCRPC and metastatic castration-sensitive prostate cancer (mCSPC).

Another advanced AR-targeting agent, BMS-986365 (Gridegalutamide), developed by BMS, employs a dual mechanism of action that combines AR degradation with receptor antagonism (NCT06764485). This dual strategy provides a more robust and durable suppression of the AR pathway compared to traditional antagonists [25].

In early-phase studies, BMS-986365 was administered to heavily pretreated patients with mCRPC. The median radiographic progression-free survival (rPFS) reached an impressive 16.5 months among chemotherapy-naïve patients, compared to just 5.5 months in those who had undergone prior chemotherapy. Furthermore, clinical benefits were seen across a wide range of patients, regardless of their AR ligand-binding domain mutational status [25,26].

Building on this promising foundation, the ongoing Phase 3 study titled rechARge is set to investigate the efficacy and safety of BMS-986365 in comparison to the investigator’s choice of therapy, including docetaxel, abiraterone plus prednisone/prednisolone, or enzalutamide, in mCRPC patients. The primary goal of this pivotal trial is to assess rPFS, reinforcing our commitment to advancing treatment options for patients in need [27].

**Scheme 1:**
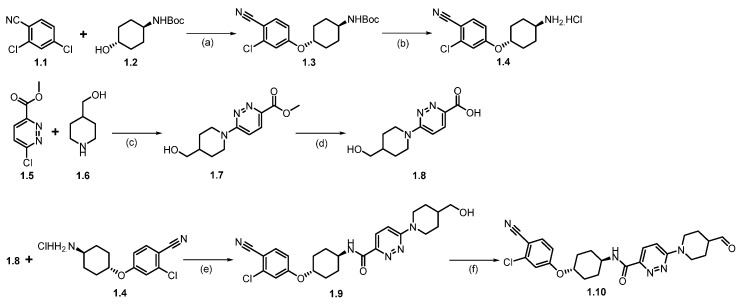
Synthesis of aldehyde fragment **1.10** of ARV-110: (**a**) NaH, DMA, 0 °C to 45 °C, 2 h; (**b**) HCl in MeOH, r.t., 2 h; (**c**) DIPEA, DMSO, 90 °C, 2 h; (**d**) LiOH.H_2_O, THF, MeOH, r.t., 1 h; (**e**) HOPO, EDC.HCl, DMA, DIPEA, 20 °C, 12 h; (**f**) TEMPO, NaOCl, NaHCO_3_, DCM, 20 °C, 12 h.

Synthesis of **ARV-110** (**1**)**.** The synthesis commenced with nucleophilic aromatic substitution (S_N_Ar) reaction between commercially available 2,4-dichlorobenzonitrile (**1.1**) and *tert*-butyl((1*R*,4*R*)-4-hydroxycyclohexyl)carbamate (**1.2**) in dimethylacetamide (DMA), using sodium hydride (NaH), which afforded ether product **1.3** (Scheme 1). In the subsequent step, the deprotection of the -Boc group from **1.3** was achieved by treating with HCl in methanol at room temperature, which afforded **1.4**. The resulting product was recrystallized from methyl *tert*-butyl ether, yielding pure product **1.4** as the HCl salt. Following this, a nucleophilic aromatic substitution (S_N_Ar) reaction was performed between compounds **1.5** and **1.6** in dimethyl sulfoxide (DMSO) with diisopropyl ethylamine (DIPEA) to form substituted product **1.7**, which was then hydrolyzed to give the acid **1.8**. The amidation of acid **1.8** with amine **1.4**, using 1-Ethyl-3-(3-dimethylaminopropyl)carbodiimide (EDC.HCl) and 2-hydroxy pyridine oxide (HOPO), yields product **1.9**. Finally, (2,2,6,6-Tetramethylpiperidin-1-yl)oxyl (TEMPO) catalyzed oxidation of primary alcohol on **1.9** with sodium hypochlorite (NaOCl) and sodium bicarbonate (NaHCO_3_) in dichloromethane (DCM) produced the key aldehyde fragment **1.10** [28].

**Scheme 2:**
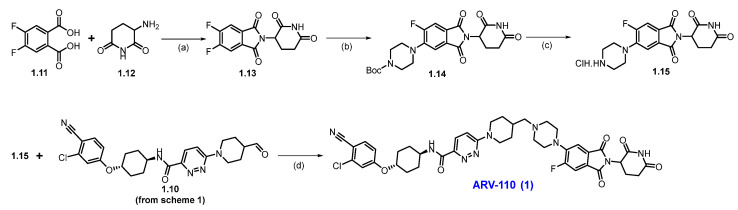
Synthesis of **ARV-110 (1):** (**a**) Ac_2_O, CH_3_COONa, 120 °C, 4 h; (**b**) Boc-piperazine, NaHCO_3_, NMP, 90 °C, 16 h; (**c**) 3M HCl in MeOH, DCM, 35 °C, 21 h; (**d**) Na(OAc)_3_BH, NMP, r.t., 3 h.

Scheme 2 outlines the final synthesis of ARV-110. Reaction of commercially available 4,5-difluorophthalic acid **1.11** and 3-amino piperidine-2,6-dione **1.12** in acetic acid and sodium acetate (NaOAc), afforded thalidomide-5,6-F **1.13**. The next step involved an S_N_Ar reaction with substituted *N*-boc piperazine scaffold **1.13** and commercially available Boc-piperazine in *N*-methyl-2-pyrrolidone (NMP) with NaHCO_3_ at 90 °C, producing *N*-Boc piperazine substituted product **1.14**. Deprotection of the *tert*-butyloxycarbonyl (Boc) group on **1.14** was carried out with HCl in methanol, giving thalidomide product **1.15** as an HCl salt, which was then recrystallized from isopropanol (IPA). Reductive amination reaction of **1.15** with aldehyde **1.10** (Scheme 1) in DMA using sodium triacetoxyborohydride (Na(OAc)_3_BH) as a reductant, resulting in crude ARV-110. Crystallization from an ethanol solution afforded **ARV-110** (**1**) as a light yellow crystalline solid [28].

**Scheme 3:**
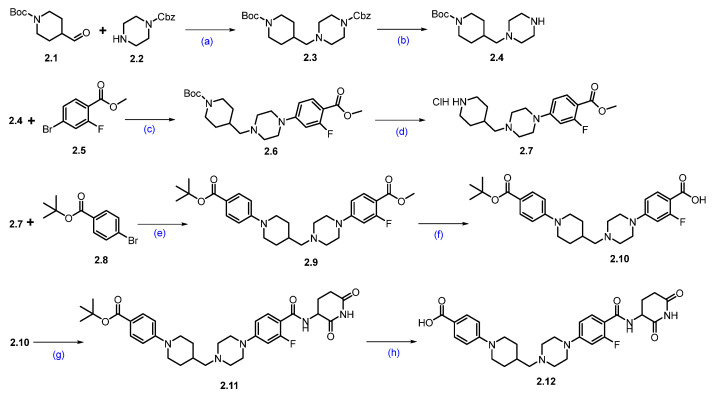
Synthesis of fragment **2.12** of **ARV-766 (2):** (**a**) NaCNBH_3_, MeOH, r.t., 10 h; (**b**) 10%Pd/C, H_2_, EtOH, 50 °C, 12 h; (**c**) Pd(OAc)_2_, BINAP, Cs_2_CO_3_, dioxane, 110 °C, 16 h; (**d**) 4N HCl in dioxane, EtOAc, 1 h; (**e**) Pd(OAc)_2_, BINAP, Cs_2_CO_3_, dioxane, 110 °C, 16 h; (**f**) LiOH.H_2_O, MeOH, H_2_O, 40 °C, 10 h; (**g**) 3-aminopiperidine-2,6-dione, EDC.HCl, HOBt, DIPEA, DMF, r.t., 10 h; (**h**) 4N HCl in dioxane, EtOAc, 1 h.

**Scheme 4:**
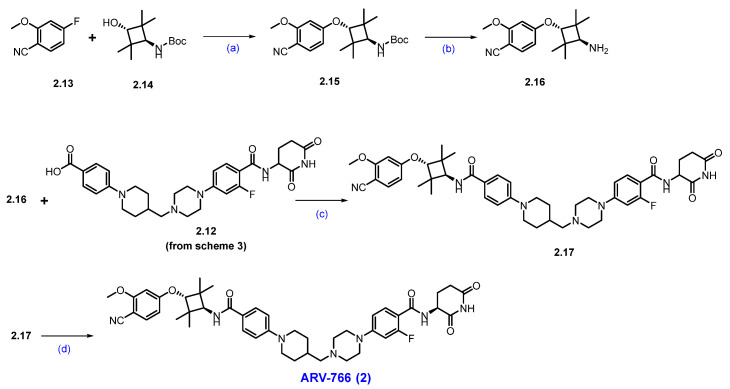
Synthesis of **ARV-766 (2):** (**a**) NaH, DMF, 0 °C to 20 °C, 4 h; (**b**) 4N HCl in dioxane, DCM, 1 h; (**c**) EDC.HCl, HOBt, DIPEA, DMF, r.t., 24 h; (**d**) SFC purification.

Synthesis of **ARV-766** (**2**). Scheme 3 and Scheme 4 illustrate the synthesis of ARV-766. The synthesis started with commercially available aldehyde **2.1** and amine **2.2**. The reductive amination of compounds **2.1** and **2.2** afforded reductive alkylated product **2.3**, and subsequent deprotection of the benzyloxycarbonyl (Cbz) group using palladium on carbon (Pd/C) in ethanol (EtOH) afforded -boc protected piperazine amine compound **2.4**. Under Buchwald coupling conditions, the reaction between amine **2.4** and aryl bromo derivative **2.5** afforded **2.6**. Following this, Boc deprotection was carried out using 4N HCl in dioxane in ethyl acetate (EtOAc) to generate scaffold **2.7**. A second Buchwald coupling reaction between piperidine amine, **2.7**, and substituted bromobenzene, **2.8** resulted in the formation of the coupled compound **2.9**, which, after being subjected to saponification with Lithium hydroxide (LiOH), produced the acid compound **2.10**. Subsequently, acid-amine coupling of **2.10** and 3-aminopiperidine-2,6-dione in the presence of EDC.HCl in DMF yielded amide compound **2.11** and deprotection of the *tert*-butyl group using 4N HCl in dioxane in EtOAc to afford key carboxylic acid compound **2.12** (Scheme 3).

The final synthesis of ARV-766 is described in Scheme 4. The S_N_Ar reaction of commercially available compounds **2.13** and **2.14**, in the presence of NaH in dimethylformamide (DMF), produced ether product **2.15** in 64% yield. Boc deprotection of **2.15** using 4*N* HCl in dioxane-furnished amine **2.16** at a quantitative yield. In the final step of the synthesis, acid **2.12** (Scheme 3) was coupled with amine **2.16** in the presence of EDC.HCl in DMF to furnish ARV-766, as a diastereomeric mixture, it was separated by SFC to obtain enantiopure **ARV-766 (2)** [14].

**Scheme 5:**
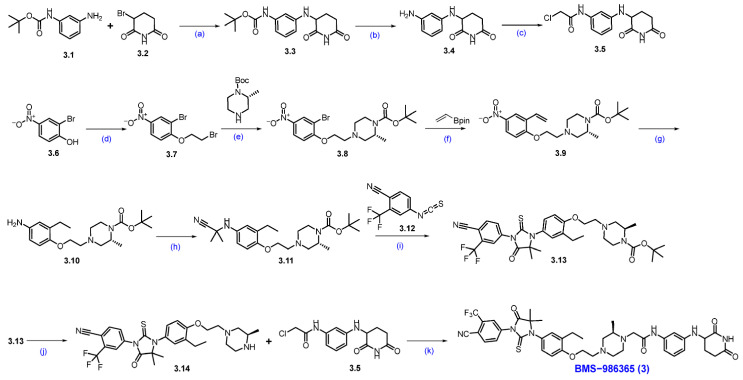
Synthesis of **BMS−986365 (3):** (**a**) NaHCO_3_, DMF, 80 °C, 16 h; (**b**) 4N HCl in dioxane, DCM, 25 °C, 30 min.; (**c**) 2-Cl-acetic acid, HATU, DIPEA, DMF, 15 °C, 1 h; (**d**) 1,2-dibromoethane, DIPEA, DMF, 60 °C, 12 h; (**e**) DIPEA, DMF, 60 °C, 12 h; (**f**) Pd(dppf)Cl_2_, K_3_PO_4_, dioxane, H_2_O, 90 °C, 12 h; (**g**) Pd/C, H_2_, MeOH, 50 psi, 30 °C, 12 h; (**h**) 2-hydroxy-2-methylpropanenitrile, MgSO_4_, 60 °C, 12 h; (**i**) DMF, r.t. for 1 h then HCl in MeOH, 80 °C, 1 h; (**j**) 4N HCl in dioxane, DCM, r.t., 2 h; (**k**) DIPEA, DMF, 60 °C, 12 h.

Synthesis of **BMS−986365** (**3**). The synthesis started with commercially available compounds *tert*-butyl (3-aminophenyl)carbamate **3.1** and 3-bromopiperidine-2,6-dione **3.2** [29]. Nucleophilic substitution reaction of **3.1** with **3.2** gave amine substituted product **3.3**. Deprotection of the Boc group of compound **3.3** with 4N HCl furnished compound **3.4** (Scheme 5). Subsequently, amidation reaction using 2-chloroacetic acid with **3.4** afforded the compound **3.5**. The synthesis of another part of the molecule began with 2-bromo-4-nitrophenol **3.6**, which was converted to compound **3.7** by following a substitution reaction with 1,2-dibromoethane. Compound **3.7** was then subjected to another substitution reaction with *tert*-butyl-(*R*)-2-methylpiperazine-1-carboxylate in the presence of DIPEA to furnish compound **3.8**. Suzuki coupling reaction of compound **3.8** with vinyl boronic acid pinacol ester by using Pd(dppf)Cl_2_ and K_3_PO_4_ furnished compound **3.9**. Nitro functional group and vinyl moiety in compound **3.9** were reduced in hydrogenation conditions using Pd/C in methanol to furnish compound **3.10**. Compound **3.10** was next converted to compound **3.11** by an addition reaction with 2-hydroxy-2-methylpropanenitrile in the presence of MgSO_4_, which gave **3.11**, which was immediately converted to compound **3.13** following another addition reaction with compound **3.12** in an acidic medium. Deprotection of the Boc group of compound **3.13** using 4N HCl furnished compound **3.14**. A substitution reaction of compound **3.14** with compound **3.5** in the presence of DIPEA in DMF furnished the final compound **BMS−986365 (3)**.

### 2.2. PROTACS Targeting Estrogen Receptor (ER)

Breast cancer is one of the most common cancers worldwide and the leading cause of cancer-related death among women. In 2023, about 297,790 U.S. women were expected to be diagnosed with invasive breast cancer, with an estimated 43,700 deaths. However, advances in treatment from 1989 to 2020 reduced mortality by 43%, saving around 460,000 lives [30]. Luminal A and luminal B classifications, accounting for 70% of cases, are defined by ER expression, making it a crucial diagnostic and therapeutic target [31]. Standard treatments for ER-positive breast cancers involve estrogen deprivation or direct ER inhibition with selective estrogen receptor modulators (SERMs) such as tamoxifen, or selective estrogen receptor degraders (SERDs), such as Fulvestrant. Tamoxifen is widely prescribed but exhibits tissue-specific effects and can act as a partial ER agonist in some contexts, potentially increasing the risk of secondary cancers with prolonged use [32]. Fulvestrant blocks ER function and promotes its degradation but is limited by poor pharmacokinetic properties and suboptimal target coverage [33].

Therapeutic resistance remains a significant obstacle, affecting 30–50% of patients with advanced metastatic breast cancer. Resistance can arise through various mechanisms, including ligand-independent ER activation via the PI3K/AKT/mTOR [34,35] and RAS/RAF/MEK/ERK [36] signaling pathways, or through gain-of-function mutations in the ESR1 gene. The most frequently observed ESR1 mutations Y537S and D538G occur in the ligand-binding domain and drive estrogen-independent ER activity [37,38]. These mutations reduce the effectiveness of aromatase inhibitors, SERMs, and SERDs. Endocrine resistance can also arise from altered interactions between ER and its coactivators or corepressors, or through compensatory signaling crosstalk between ER and growth factor receptor pathways [39,40].

Consequently, there is a growing interest in the development of next-generation therapies, including orally bioavailable PROTAC-based ER degraders. These agents aim to achieve more complete ER suppression and overcome resistance associated with receptor mutations that confer agonist activity [41,42,43,44]. Notable examples include Arvinas’s PROTAC-based degrader Vepdegestrant. Additional ER-targeting PROTACs, such as AC682 and HRS-1358, are currently in clinical trials, although their molecular structures have not been disclosed [45].

Clinical Development and Outcomes of Vepdegestrant **(ARV-471, 4):** Data from the 2025 ASCO Annual Meeting, derived from the Phase 3 VERITAC-2 trial (NCT05654623), indicate that Vepdegestrant (ARV-471) provides a statistically significant and clinically meaningful improvement in progression-free survival (PFS) compared to Fulvestrant in previously treated patients with ER+/HER2- breast cancer. The median follow-up was 7.4 months for the Vepdegestrant group (*n* = 136) and 6.0 months for the Fulvestrant group (*n* = 134). Median PFS was 5.0 months with Vepdegestrant and 2.1 months with Fulvestrant. At the time of analysis, 58% of PFS events had occurred in the Vepdegestrant arm compared to 71% in the Fulvestrant arm, with ongoing treatment in 33% and 12% of patients, respectively. The six-month PFS rates were 45.2% for Vepdegestrant and 22.7% for Fulvestrant. However, the difference in PFS was less pronounced among the overall study population, with a median PFS of 3.8 months for Vepdegestrant and 3.6 months for Fulvestrant. In the ESR1-mutant subgroup, Vepdegestrant (*n* = 121) more than doubled the clinical benefit rate (CBR) compared with Fulvestrant (*n* = 119) at 42.1% versus 20.2%, respectively. The objective response rate (ORR) was also higher with Vepdegestrant, at 18.6% (*n* = 97), compared to 4.0% with Fulvestrant. Safety data from all treated patients (Vepdegestrant, *n* = 312; Fulvestrant, *n* = 307) indicated that both treatments were generally well tolerated [5,46,47].

**Scheme 6:**
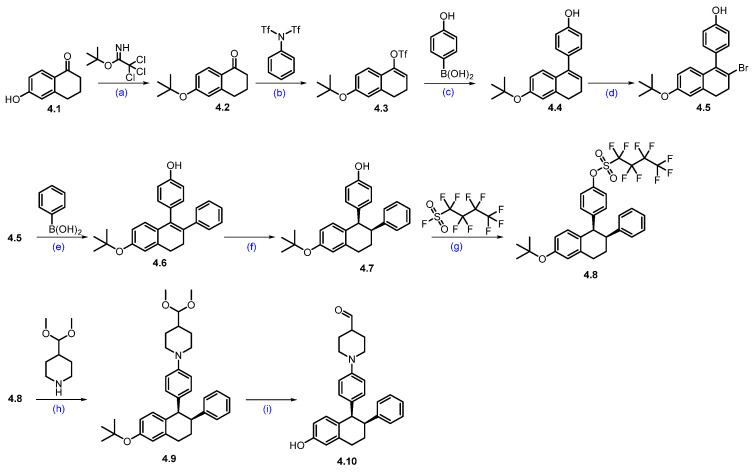
Synthesis of aldehyde fragment **4.10** of **ARV-471 (4):** (**a**) PPTS, DCM, 10 °C, 16 h; (**b**) LDA, THF, −70 °C, 1 h then 20 °C for 2 h; (**c**) Pd(dppf)Cl_2_, K_2_CO_3_, dioxane, H_2_O, 100 °C, 10 h; (**d**) NBS, ACN; r.t., 1.5 h; (**e**) Pd(dppf)Cl_2_, K_2_CO_3_, dioxane, H_2_O, 100 °C, 12 h; (**f**) H_2_, Pd/C, 50 psi, 30 °C, 36 h then chiral SFC; (**g**) K_2_CO_3_, THF, ACN, r.t., 16 h; (**h**) Pd(OAc)_2_, X-phos, NaO*^t^*Bu, toluene, 90 °C, 16 h; (**i**) 2M H_2_SO_4_, THF, 70 °C, 1 h.

**Scheme 7:**
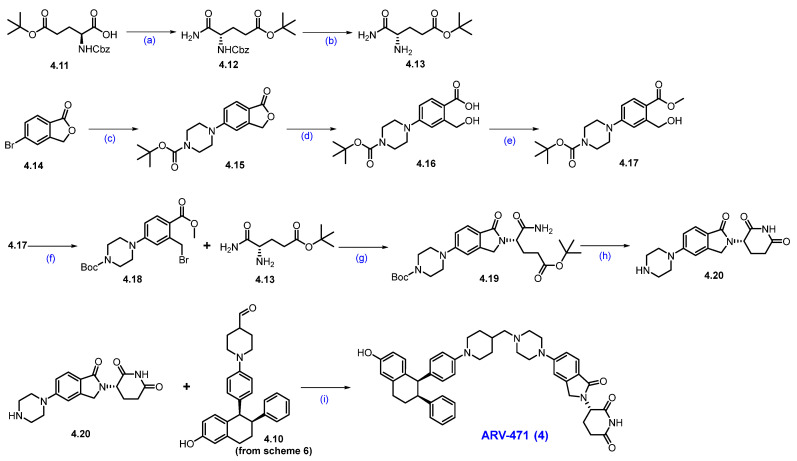
Synthesis of **ARV-471 (4):** (**a**) pyridine, (Boc)_2_O, NH_4_HCO_3_, 1,4-dioxane, 0 °C to 25 °C, 16 h; (**b**) 10%Pd/C, H_2_, MeOH, 50 psi, r.t., 16 h; (**c**) Pd_2_(dba)_3_, X-phos, K_3_PO_4_, 1,4-dioxane, 100 °C, 15 h; (**d**) NaOH, THF. MeOH, H_2_O, r.t., 16 h; (**e**) TMS-diazomethane, MeOH/EtOAc, −10 °C, 15 min; (**f**) CBr_4_, PPh_3_, THF; r.t., 1 h; (**g**) DIPEA, ACN, 80 °C, 12 h; (**h**) benzenesulfonic acid, ACN, 85 °C, 12 h; (**i**) NaCNBH_3_, NaOAc, MeOH, 1,2-DCE, 20 °C, 12 h.

Synthesis of **ARV-471 (4)** [48]. The synthesis of ARV-471 was shown in Scheme 6 and Scheme 7. The commercially available phenolic hydroxyl group of 6-hydroxy-1-tetralone **4.1** was treated with *tert*-butyl 2,2,2-trichloroacetimidate in DCM, which afforded *tert*-butyl ether derivative **4.2**. The ketone in **4.2** was converted into triflate **4.3** with LDA at low temperature to form an enolate, which then reacted with phenyl triflimide, yielding **4.3** in 78% yield. The reactive triflate scaffold underwent a palladium-catalyzed Suzuki–Miyaura coupling with (4-hydroxyphenyl)boronic acid to afford product **4.4** with an 88% yield. Vinylic bromination of **4.4** using NBS in acetonitrile produced the key bromo derivative **4.5**. The resultant brominated compound **4.5** was subsequently used in a palladium-catalyzed Suzuki–Miyaura coupling using phenylboronic acid, generating coupled compound **4.6**. Reduction of the double bond with Pd/C, followed by chiral SFC separation, yielded the desired syn-disubstituted tetrahydronaphthalene **4.7**. The phenolic hydroxyl group in **4.7** was further activated with perfluorobutylsulfonyl fluoride in the presence of K_2_CO_3_ in a mixture of THF and acetonitrile, obtaining **4.8** with a 91% yield. Subsequently, the palladium-catalyzed Buchwald–Hartwig amination coupling of nonaflate **4.8** with 4-(dimethoxymethyl)piperidine proceeded efficiently using Pd(OAc)_2_ as the catalyst, XPhos as the ligand, and *t*-BuONa as the base in toluene, yielding acetal **4.9** in an excellent 87% yield. Acetal **4.9** was effectively deprotected with 2M H_2_SO_4_ in THF, affording aldehyde 4.10 in 92% yield [49,50].

Next, the commercially available acid **4.11** was converted into the amide derivative **4.12** using ammonium bicarbonate (NH_4_HCO_3_), Boc anhydride ((Boc)_2_O), and pyridine in 1,4-dioxane. The -Cbz group in **4.12** was then removed using Pd/C in MeOH, resulting in amine **4.13**. Next, commercially available 5-bromophthalide **4.14** and Boc-piperazine underwent Buchwald coupling to produce compound **4.15**. This compound was hydrolyzed with NaOH to yield carboxylic acid derivative **4.16**, which was subsequently converted to ester **4.17** using TMS-diazomethane (Scheme 7).

The benzylic alcohol in compound **4.17** was subjected to an Appel reaction using CBr_4_ and PPh_3_ in THF, resulting in the formation of the benzylic bromide scaffold **4.18**. Aliphatic amine derivative **4.13** was then reacted with **4.18** in acetonitrile in the presence of DIPEA to yield compound **4.19**. In situ cyclization and -Boc deprotection of **4.19** were achieved by heating it in acetonitrile with benzenesulfonic acid, resulting in the key scaffold amine **4.20** as a benzenesulfonic acid salt. Finally, a reductive amination between **4.20** and **4.10** (Scheme 6) in the presence of NaOAc and sodium cyanoborohydride (NaCNBH_3_) produced **ARV-471 (4)** with a yield of 35% [48].

### 2.3. PROTACS Targeting Bromodomain-Containing Protein 9 (BRD9)

Synovial sarcoma is a rare, aggressive cancer mainly affecting young men under 30, with about 1000 cases annually. It usually arises near large joints in the limbs. While therapies targeting BRD9 have been explored, conventional inhibition has been ineffective due to BRD9′s role as a scaffolding protein in complex cellular processes. Targeted degradation of BRD9 presents a promising therapeutic alternative. While classical inhibitors that disrupt BRD9′s have yielded limited phenotypic responses, genetic knockdown has shown more profound effects, underscoring the limitations of traditional small-molecule inhibitors. A key challenge is that epigenetic regulators like BRD9 typically possess multi-domain architectures and function within multi-subunit chromatin remodeling complexes, which diminishes the impact of bromodomain inhibition alone. As a result, these inhibitors do not completely shut down the oncogenic transcription driven by BRD9 [51,52]. Importantly, both synovial sarcoma and SMARCB1-deficient tumors exhibit a critical dependence on BRD9, a core component of the noncanonical SWI/SNF complex that includes the SS18-SSX fusion oncoprotein. This creates a synthetic lethal vulnerability: cancer cells harboring the SS18-SSX fusion require BRD9 for oncogenic transcription and proliferation, whereas normal tissues show minimal dependence on BRD9 [53]. As a result, targeted degradation of BRD9 represents a promising therapeutic strategy, selectively eliminating cancer cells through exploitation of this synthetic lethal dependency [54,55,56].

Clinical Development and Outcomes of **CFT8634 (5)** and **FHD-609 (6):** Two promising BRD9 PROTACs, CFT8634 and FHD-609, have advanced into clinical trials. They are designed to degrade BRD9 specifically for the treatment of advanced and metastatic synovial sarcoma, ensuring selectivity over BRD7. However, in April 2023, Foghorn Therapeutics suspended patient enrollment in the FHD-609 trial after a participant experienced a serious grade 4 QTc prolongation at the second-highest dose. Patients affected by this event have been transitioned to a reduced dose, while the FDA has placed the study under a partial clinical hold, allowing those who are responding to the treatment to continue (NCT04965753) [57]. C4 Therapeutics has also introduced an oral BRD9 degrader, CFT8634, intended for patients with synovial sarcoma and SMARCB1-null solid tumors (NCT05355753). Despite achieving high levels of BRD9 degradation in the Phase 1 clinical trial, further investigation was halted in November 2023 due to inadequate efficacy as a standalone therapy [58,59].

**Scheme 8:**
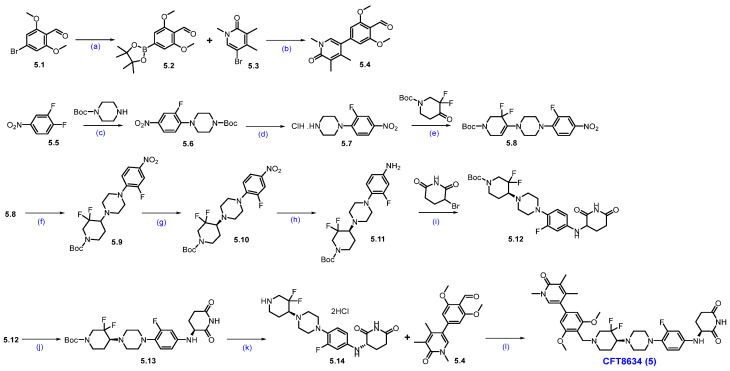
Synthesis of **CFT8634 (5):** (**a**) B_2_(Pin)_2_, KOAc, Pd(dppf)Cl_2_, dioxane, 140 °C, 40 min.; (**b**) K_2_CO_3_ (aq. sol.), 120 °C, MW irradiation, 30 min.; (**c**) Cs_2_CO_3_, DMF, r.t., 16 h; (**d**) 4M HCl in dioxane, r.t., 2 h; (**e**) NaOAc, AcOH, ACN, 135 °C, 12 h; (**f**) NaCNBH_3_, 1,2-DCE/MeOH, AcOH, r.t., 24 h; (**g**) SFC separation (**h**) 10% Pd/C/H_2_, EtOAc; (**i**) NaHCO_3_, DMF, 70 °C; (**j**) Chiral HPLC separation; (**k**) HCl in *^i^*PrOAc, r.t., 10 h; (**l**) Na(OAc)_3_BH, AcOH, DIPEA, DMAc, −5 °C to r.t., 16 h.

Synthesis of **CFT-8634 (5):** Scheme 8 outlines the synthesis of CFT8634 as disclosed in the patent application by C4 Therapeutics [60]. The synthetic route begins with aldehyde **5.1**, which underwent a Suzuki coupling reaction with bis(pinacolato)diboron to yield boronate ester **5.2**. The aryl boronate scaffold is then coupled with aryl bromo compound **5.3** via another Suzuki reaction to produce di-aryl compound **5.4**. In parallel, 1,2-difluoro-4-nitrobenzene (**5.5**) reacted with Boc-piperazine in a nucleophilic substitution to give *N*-Boc-piperazine substituted product **5.6**, which was subsequently Boc-deprotected to form amine **5.7** as a HCl salt. Reductive amination of amine **5.7** with *tert*-butyl 3,3-difluoro-4-oxopiperidine-1-carboxylate, with NaCHBH_3_, produces derivative **5.9**. A chiral resolution of **5.9** yielded the enantiomerically pure compound **5.10**. The nitro group in **5.10** was reduced using hydrogen gas and palladium on carbon, generating aryl amine **5.11**. Subsequent nucleophilic substitution of amine **5.11** with a Bromo piperidinedione affords compound **5.12**. Chiral chromatographic separation then isolates the desired diastereomer **5.13**. Final Boc deprotection of **5.13** using HCl in isopropyl acetate generates amine **5.14**, which underwent reductive amination with aldehyde **5.4** to yield the final product, **CFT8634** (**5**).

**Scheme 9:**
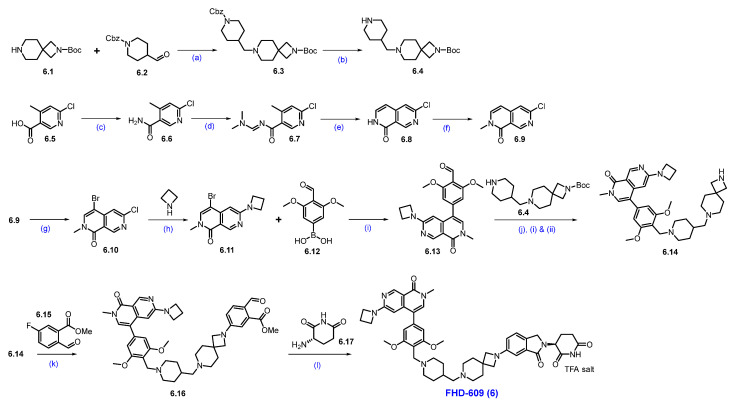
Synthesis of **FHD-609 (6):** (**a**) Na(OAc)_3_BH, NaOAc, MeOH, r.t.; (**b**) Pd/C, H_2_, MeOH, 40 psi, r.t., 16 h; (**c**) NH_4_Cl, HATU, DIPEA, DCM, r.t., 3 h; (**d**) DMF-DMA, 2-Me-THF, 80 °C, 1 h; (**e**) *t*-BuOK, THF, 60 °C, 30 min.; (**f**) NaH, MeI, THF, 0 °C to r.t., 12 h; (**g**) NBS, DMF, 90 °C, 2 h; (**h**) K_2_CO_3_, DMSO, 130 °C, 2 h; (**i**) Pd(dppf)Cl_2_, Cs_2_CO_3_, dioxane, H_2_O, 70 °C, 2 h; (**j**) (i) Na(OAc)_3_BH, NaOAc, NMP, MeOH; (ii) 5% H_2_SO_4,_ heating; (**k**) Na_2_CO_3_, DMA, reflux, 16 h; (**l**) NaCNBH_3_, AcOH, THF/MeOH, 0 °C to r.t., 16 h.

Synthesis of **FHD-609 (6):** Scheme 9 describes the synthesis of FHD-609 [61]. The synthesis started with a reductive amination reaction of amine **6.1** with aldehyde **6.2** to produce **6.3**. The Cbz group in **6.3** was deprotected under hydrogenation conditions, affording free amine **6.4**. Separately, acid **6.5** is converted to amide **6.6** through an amidation reaction using ammonium chloride. Formation of an imine **6.6** from compound **6.7**, followed by cyclization under strongly basic conditions, yielded Cl-naphthyridone derivative **6.8**. An *N*-methylation reaction of **6.8** with sodium hydride and methyl iodide yielded compound **6.9**, which, upon bromination with NBS, furnished bromo derivative **6.10**. Substitution reaction of **6.10** with cyclobutyl amine furnished compound **6.11**. Subsequent Suzuki coupling reaction of **6.11** with the desired substituted phenylboronic acid **6.12** furnished product **6.13**. Reductive amination of **6.13** with amine **6.4**, followed by Boc deprotection, furnishes key scaffold **6.14**. An S_N_Ar reaction of **6.14** with compound **6.15** using Na_2_CO_3_ and DMA under heating conditions furnished compound **6.16**. Finally, a reductive amination of aldehyde **6.16** with amine **6.17,** followed by cyclization, completes the synthesis of **FHD-609** (**6**).

### 2.4. PROTACs Targeting B-Cell Lymphoma–Extra Large (Bcl-xL)

T-cell lymphoma (TCL) is a rare and difficult-to-diagnose hematologic malignancy that originates from T lymphocytes. These cancerous cells evade apoptosis by overexpressing anti-apoptotic members of the Bcl-2 protein family, particularly Bcl-xL. Traditional small-molecule inhibitors such as ABT263 (navitoclax) have shown limited clinical success in advanced TCL due to dose-limiting thrombocytopenia, as Bcl-xL is also essential for platelet survival. To overcome this challenge, researchers developed DT2216, a VHL-recruiting PROTAC designed to selectively degrade Bcl-xL in tumor cells while sparing platelets. This tissue-selectivity arises from the differential expression of the E3 ligase VHL: platelets express very low levels of VHL, making them less vulnerable to Bcl-xL degradation, whereas tumor cells with higher VHL expression are effectively targeted [62,63]. DT2216 has received orphan drug designation for the treatment of TCL and small cell lung cancer, as well as fast track designation for adult patients with R/R peripheral TCL (PTCL) and cutaneous TCL (CTCL). DT2216 represents a therapeutic approach, demonstrating how targeted protein degradation can leverage differential E3 ligase expressions to enable tissue-selective cancer treatment with improved safety profiles [64,65].

**Scheme 10:**
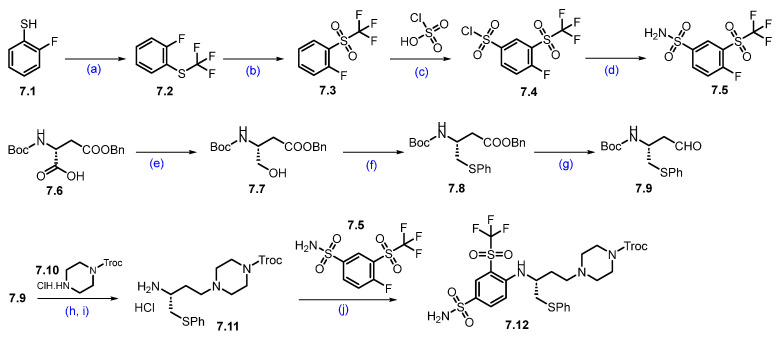
Synthesis of fragment **7.12** of **DT-2216 (7):** (**a**) CF_3_I, methyl viologen dichloride, NEt_3_, DMF, r.t., 16 h; (**b**) NaIO_4_; RuCl_3_.H_2_O, ACN, H_2_O, r.t., 3 h; (**c**) chlorosulfonic acid, 120 °C, 5 h; (**d**) Isopropyl acetate, aq. NH_3_; (**e**) N-methylmorpholine, isobutyl chloroformate, THF, −25 °C to −15 °C then NaBH_4_; (**f**) Bu_3_P, diphenyl disulfide, toluene, 80 °C, 16 h; (**g**) DIBAL-H, toluene, −78 °C, 2 h; (**h**) Na(OAc)_3_BH, NEt_3_, DCM, r.t., 1 h; (**i**) 4M HCl in dioxane, DCM, r.t., 1 h; (**j**) NEt_3_, ACN, reflux, 4 h.

**Scheme 11:**
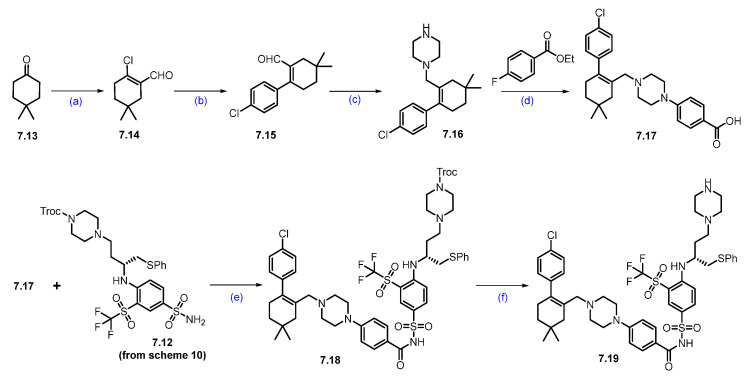
Synthesis of fragment **7.19** of **DT-2216 (7):** (**a**) POCl_3_, PBr_3_, DMF, 0 °C to 60 °C, 16 h; (**b**) 4-Cl-C_6_H_4_B(OH)_2_, PdCl_2_(PPh_3_)_2_, KOAc, 1,4-dioxane, H_2_O, 90 °C, 12 h; (**c**) NaCNBH_3_, TFA/DCM, (**d**) DABCO, LiOH; (**e**) EDC.HCl, DMAP, DCM, r.t., 16 h; (**f**) Zn, AcOH, THF, r.t., 16 h.

**Scheme 12:**
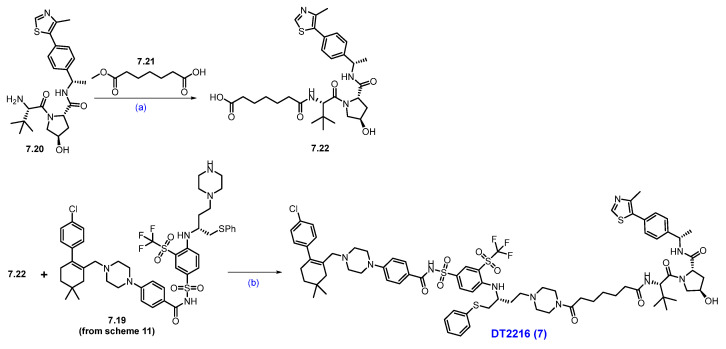
Synthesis of **DT-2216 (7):** (**a**) HATU, NEt_3_, DCM, 0 °C to r.t., 2 h; LiOH, MeOH, H_2_O, r.t., 3 h; (**b**) HATU, NEt_3_, DCM, r.t., 2 h.

Synthesis of **DT-2216 (7)**. The synthesis of DT-2216 was described in Scheme 10, Scheme 11 and Scheme 12 [66]. The synthesis was initiated from commercially available fluorothiophenol **7.1**. The first step involves trifluoromethylation of 2-fluorobenzene-1-thiol **7.1** with trifluoromethyl iodide and methyl viologen dichloride, which afforded compound **7.2**. The compound **7.2** was oxidized with periodic acid using ruthenium(III) chloride hydrate as a catalyst in a CH_3_CN:H_2_O solvent mixture, to yield compound **7.3**. In the next step, (trifluoromethyl)sulfonylbenzene derivative **7.3** reacted with chlorosulfonic acid at an elevated temperature to give the compound **7.4**, which further reacted with aqueous ammonia in isopropyl acetate (iPrOAc) to provide the compound **7.5** (Scheme 10).

Next, Boc-D-Asp(OBzl)-OH (**7.6**) was first converted to its corresponding acid chloride, which was then reduced with sodium borohydride (NaBH_4_) to afford alcohol **7.7**. The alcohol **7.7** was converted to sulfide **7.8** using diphenyl disulfide. The ester functionality in compound **7.8** was treated with DIBAL-H to furnish aldehyde **7.9**. Compound **7.9** was converted to amine **7.11** following a two-step protocol. In the first step, the aldehyde reacted with Troc-protected piperazine **7.10** under reductive amination conditions. In the next step, 4M HCl-mediated -Boc deprotection afforded compound **7.11** as a HCl salt. Amine **7.11** was reacted with **7.5** in an S_N_Ar condition to furnish product **7.12**.

As shown in Scheme 11, compound **7.19** was prepared from 4,4-dimethylcyclohexan-1-one (**7.13**) through a sequence of reactions. In the first step, the commercially available compound 4,4-dimethylcyclohexanone was treated with PBr_3_ and POCl_3_ in DMF to obtain aldehyde **7.14**, which was coupled with 4-chloro phenylboronic acid using Suzuki conditions to give compound **7.15**. The aldehyde functionality of **7.15** was reacted with Boc-piperazine under reductive amination conditions, followed by Boc-deprotection, to furnish **7.16**. The scaffold **7.16** was added to 4-fluoro ethyl benzoate in an S_N_Ar condition, followed by a saponification reaction, which furnished acid **7.17**. An EDC.HCl-mediated coupling of acid **7.17** and sulfonamide **7.12** furnished compound **7.18**. Finally, the Troc-protecting group was deprotected using Zinc (Zn) and acetic acid (AcOH) to obtain key scaffold **7.19** [66].

Next, commercially available amine **7.20** was converted to acid derivative **7.22** following a two-step protocol (Scheme 12). In the first step, HATU-mediated coupling of amine **7.20** with acid **7.21** formed an amide, and in the second step, the methyl ester was saponified using LiOH to furnish the acid **7.22**. Finally, an acid–amine coupling reaction was carried out between acid **7.22** and amine **7.19** (Scheme 11) utilizing HATU and NEt_3_ in DCM to obtain **DT-2216** (**7**) [66].

### 2.5. PROTACs Targeting Bruton’s Tyrosine Kinase (BTK)

BTK plays a vital role in B cell receptor signaling, which is essential for B cell development, function, and survival. B-cell malignancies such as chronic lymphocytic leukemia (CLL) and mantle cell lymphoma (MCL) rely heavily on BTK signaling for proliferation and survival. CLL is the most prevalent form of leukemia among adults in Western countries, accounting for roughly 30% of all leukemia cases [67]. Covalent and non-covalent Bruton’s tyrosine kinase inhibitors (BTKis), such as ibrutinib and zanubrutinib, are widely used in the treatment of B-cell malignancies. However, disease progression during BTKi therapy is often driven by the emergence of resistance mutations [68]. The most common mechanism of resistance in chronic lymphocytic leukemia (CLL) during prolonged exposure to covalent BTK inhibitors is the BTK Cys481Ser (C481S) mutation. This mutation disrupts the covalent binding required for inhibitor efficacy, rendering these therapies ineffective [69,70,71].

To address this resistance, a new generation of non-covalent BTK inhibitors (ncBTKi), including pirtobrutinib and nemtabrutinib, has been developed. These inhibitors do not rely on binding to Cys481 and have shown encouraging efficacy. However, resistance to ncBTKi can still emerge through a variety of additional BTK mutations. These include gatekeeper mutations (T474I/F/S/L/Y), mutations at the C381 residue (C381S/R/Y), kinase-impaired variants such as L528W, and mutations near the ATP-binding pocket (e.g., D539A, V416L, Y545N, and A428D) [72]. These mutations not only interfere with inhibitor binding but may also promote the recruitment of alternative kinases or confer a neomorphic scaffolding function to BTK, thereby enabling sustained oncogenic signaling despite BTK inhibition [73,74,75].

Unlike traditional inhibitors, BTK degraders eliminate both the kinase activity and the resistance-associated scaffolding function, effectively killing inhibitor-resistant cancer cells. Currently, six BTK-targeting PROTACs are undergoing clinical evaluation for B-cell malignancies. Nurix Therapeutics is at the forefront of this effort, developing two BTK degraders: NX-2127 (NCT04830137), which simultaneously degrades BTK and the transcription factor Ikaros; and NX-5948 (NCT05131022), a next-generation compound designed to selectively degrade BTK [76,77,78].

Clinical Development and Outcomes of **NX-2127 (8)**: As of 9 June 2023, a Phase 1a/b clinical trial had enrolled 47 patients with various B-cell malignancies, including chronic lymphocytic leukemia (CLL), small lymphocytic lymphoma (SLL), diffuse large B-cell lymphoma (DLBCL), mantle cell lymphoma (MCL), marginal zone lymphoma (MZL), Waldenström macroglobulinemia (WM), and follicular lymphoma (FL). The treatment demonstrated a manageable safety profile, with common adverse events (AEs) including neutropenia, hypertension, anemia, and atrial fibrillation. In terms of efficacy, among the evaluable patients with CLL/SLL, nine achieved partial responses, eleven had stable disease, and four experienced disease progression at the time of data cutoff [79]. These early findings indicate that NX-2127 may offer therapeutic benefits in patients with heavily pretreated B-cell malignancies.

Clinical Development and Outcomes of **NX-5948 (9)**: FDA has granted fast-track designation to NX-5948 for the treatment of adult patients with relapsed/refractory WM who have received at least two lines of therapy, including a BTK inhibitor [80,81,82].

Among 49 evaluable patients with relapsed/refractory CLL/small lymphocytic lymphoma (SLL), NX-5948 achieved an objective response rate (ORR) of 75.5% across all doses tested. Notably, with longer treatment duration, the ORR increased to 84.2% in patients with at least two response assessments. Regardless of prior treatments, baseline mutations, high-risk molecular features, or central nervous system (CNS) involvement, responses were observed. The treatment was well tolerated, with common adverse events including mild to moderate purpura/contusion, fatigue, petechiae, neutropenia, and rash. In the cohort of patients with relapsed/refractory Waldenström’s Macroglobulinemia (WM), NX-5948 demonstrated an ORR of 77.8%, with objective responses observed in 7 out of 9 evaluable patients. The treatment was generally well tolerated, with a safety profile consistent with previous findings. These preliminary findings suggest that NX-5948 is a promising therapeutic candidate for patients with B-cell malignancies, including those resistant to existing BTK inhibitors.

**Scheme 13:**
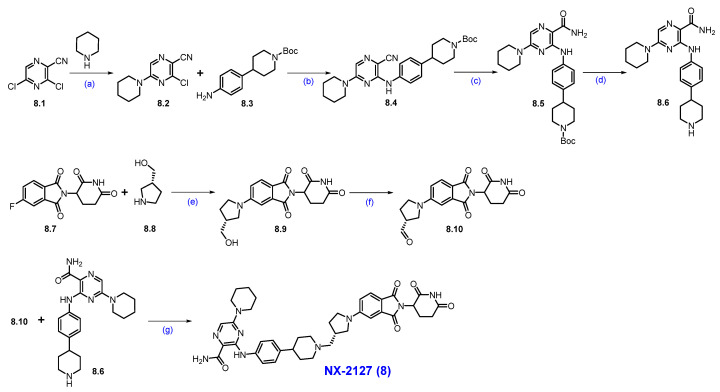
Synthesis of **NX-2127 (8):** (**a**) Piperidine, DIPEA, DMF, r.t., 2 h; (**b**) BINAP, Pd(OAc)_2_, Cs_2_CO_3_, dioxane, 90 °C, 1.5 h; (**c**) H_2_O_2_, NaOH, MeOH, DMSO, r.t, 30 min.; (**d**) TFA, DCM, r.t., 2 h; (**e**) DIPEA, NMP, 80 °C, 12 h; (**f**) DMP, DCM, r.t., 16 h; (**g**) Na(OAc)_3_BH, DIPEA, dioxane, r.t., 16 h.

Synthesis of **NX-2127 (8):** The synthesis of **NX-2127** was shown in Scheme 13 [78]. S_N_Ar reaction of commercially available 3,5-dichloropyrazine-2-carbonitrile **8.1** and piperidine in DMF under heating conditions, which afforded product **8.2**. It was then subjected to a Buchwald coupling reaction with compound **8.3** to furnish product **8.4**. The cyano functionality on **8.4** was oxidized with H_2_O_2_/NaOH to afford **8.5**. Deprotection of the Boc group in **8.5** using trifluoroacetic acid (TFA) in DCM yielded amine scaffold **8.6**. In parallel, an S_N_Ar reaction of commercially available 5-fluoro thalidomide **8.7** with alcohol **8.8** in the presence of DIPEA in NMP to furnish pyrrolidine substituted scaffold **8.9**. Compound **8.9** was then oxidized to aldehyde **8.10** using Dess-Martin reagent (DMP) in DCM. Finally, a reductive amination reaction between aldehyde **8.10** and amine **8.6** furnished compound **NX-2127 (8)**.

**Scheme 14:**
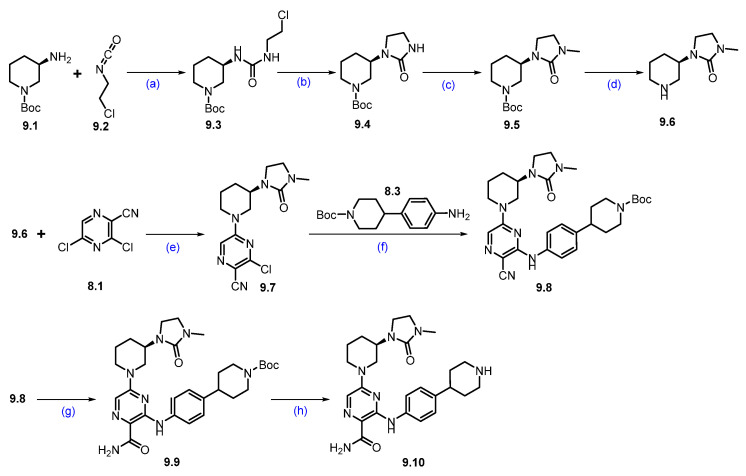
Synthesis of fragment **9.10** of **NX-5948 (9)**: (**a**) NEt_3_, DCM, 0 °C to r.t., 3 h; (**b**) NaH, THF, 0 °C to r.t., 10 h; (**c**) NaH, MeI, THF, 0 °C to r.t., 16 h; (**d**) 4N HCl in dioxane, r.t., 2 h; (**e**) DIPEA, DMF, 0 °C to r.t., 16 h; (**f**) Pd(OAc)_2_, BINAP, Cs_2_CO_3_, 110 °C, 3 h; (**g**) H_2_O_2_, Cs_2_CO_3_, MeOH, DMSO, 0 °C to r.t., 2 h; (**h**) TFA in DCM, r.t., 30 min.

**Scheme 15:**
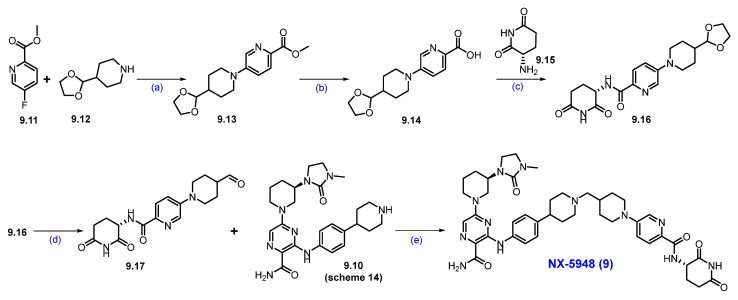
Synthesis of **NX-5948 (9):** (**a**) DIPEA, DMSO, 100 °C, 14 h; (**b**) NaOH, THF, H_2_O, r.t., 16 h; (**c**) HATU, DIPEA, DMF, r.t., 24 h; (**d**) 2M HCl in THF, r.t., (**e**) Na(OAc)_3_BH, AcOH, 1,2-DCE, r.t., 16 h.

Synthesis of **NX-5948 (9)**: The synthesis of NX-5948 was shown in Scheme 14 and Scheme 15 [83]. The first step, commercially available *tert*-butyl-(*R*)-3-aminopiperidine-1-carboxylate **9.1** and 1-chloro-2-isocyanatoethane **9.2** were reacted in the presence of triethylamine in DCM to furnish compound **9.3**. Intramolecular S_N_2 reaction of compound **9.3** in the presence of NaH produced cyclized moiety **9.4**. *N*-methylation of compound **9.4** with methyl iodide yielded compound **9.5**. The Boc protecting group in **9.5** was removed using HCl in dioxane, yielding the free amine **9.6**. Next, an aromatic nucleophilic substitution between compound **8.1** and amine **9.6** in the presence of diisopropylethylamine (DIPEA) in DMF yielded derivative **9.7**. Subsequently, Buchwald amination of **9.7** with amine **8.3**, using Pd(OAc)_2_ and BINAP as the catalytic system, provided compound **9.8**. Oxidation of the cyano group in **9.8** with hydrogen peroxide and cesium carbonate (Cs_2_CO_3_) afforded compound **9.9**. Finally, Boc deprotection of **9.9** using TFA in DCM furnished the key scaffold amine **9.10**.

An aromatic nucleophilic substitution reaction was carried out between commercially available methyl-5-fluoropicolinate **9.11** and 4-(1,3-dioxolan-2-yl)piperidine **9.12** at elevated temperatures in DMSO using DIPEA as the base, affording piperidine-substituted product **9.13**. Saponification of **9.13** with sodium hydroxide in a THF/water mixture yielded carboxylic acid **9.14**. This derivative was then coupled with commercially available (*S*)-3-aminopiperidine-2,6-dione **9.15** using HATU and DIPEA in DMF to generate compound **9.16**. Subsequent acid-catalyzed hydrolysis of the acetal group in **9.16** with 2M HCl in THF afforded aldehyde **9.17**. Finally, reductive amination between aldehyde **9.17** and amine **9.10** (Scheme 14) successfully furnished the final product, **NX-5948** (**9**).

### 2.6. PROTACs Targeting Signal Transducer and Activator of Transcription 3 (STAT3)

Signal transducer and activator of transcription 3 (STAT3) is a transcription factor that serves as a promising therapeutic target for cancer and other diseases. Among all STAT proteins, STAT3 has attracted significant attention for its roles in cell growth, survival, differentiation, regeneration, immune response, and cellular respiration [84]. Persistent activation of STAT3 contributes to oncogenesis within the tumor microenvironment, and high Phospho-STAT3 activation is associated with worse outcomes in cancers such as non-small cell lung cancer, gastric cancer, and colorectal cancer. In addition to its involvement in cancer, STAT3 is also implicated in autoimmune and inflammatory diseases such as rheumatoid arthritis, Crohn’s disease, atherosclerosis, and inflammatory bowel disease [85,86].

However, targeting STAT3 with inhibitors has proven challenging due to the highly charged phosphotyrosine binding site located in its SH2 domain. A significant challenge with SH2 domain inhibitors is the high degree of homology STAT3 shares with other STAT family members, making it challenging to achieve selectivity. Moreover, STAT3 can exhibit transcriptional activity in its monomeric form, so inhibitors that only block dimerization may provide only partial suppression of its gene-regulatory functions. To overcome these limitations, PROTACs have emerged as a promising strategy for targeting STAT3 [87,88,89,90]. For instance, the STAT3 degrader SD-36 has demonstrated over 1000-fold greater potency in suppressing STAT3 activity compared to its parent compound SI-109. Notably, while SI-109 binds STAT1 and STAT4 with similar affinity, SD-36 selectively degrades STAT3. This highlights a key advantage of degraders: cellular efficacy can be achieved even when biochemical affinity is modest [91].

Clinical Development and Outcomes of **KT-333 (10):** KT-333 is a first-in-class, potent, and highly selective PROTAC degrader of STAT3 developed by Kymera Therapeutics that has progressed into a clinical trial. It is undergoing Phase 1a/1b trials for relapsed/refractory classic Hodgkin’s lymphoma (cHL), cutaneous T-cell lymphoma (CTCL), and NK-cell lymphoma (NCT05225584). KT-333 has effectively and selectively degraded STAT3 in these trials, inhibiting the STAT3 pathway and demonstrating clinically significant responses in specific patient groups [92,93,94,95].

KT-333 achieved up to 95% mean maximum STAT3 degradation in peripheral blood mononuclear cells at higher dose levels. Early clinical results have also revealed signs of antitumor activity across various hematologic malignancies. Notably, two patients with relapsed/refractory Hodgkin’s lymphoma achieved complete responses and subsequently underwent potentially curative stem cell transplants. In addition, patients with cutaneous T-cell lymphoma (CTCL) experienced substantial tumor reductions. These preliminary outcomes suggest that KT-333 holds promise as a transformative treatment for STAT3-driven cancers [93].

**Scheme 16:**
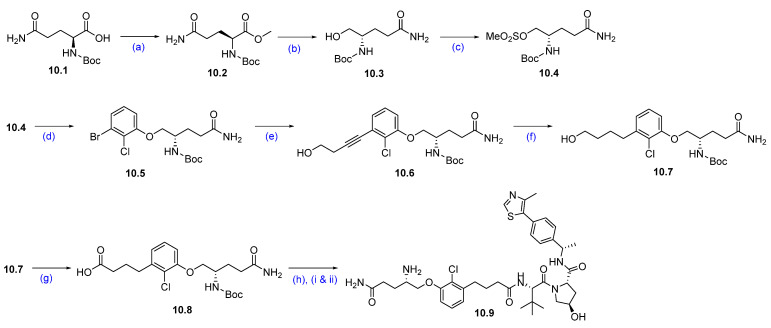
Synthesis of fragment **10.9** of **KT-333 (10):** (**a**) DCC, DMAP, DCM, MeOH, r.t., 16 h; (**b**) NaBH_4_, THF, MeOH, 0 °C to r.t., 16 h; (**c**) MsCl, NEt_3_, THF, 0 °C to r.t., 3 h; (**d**) 3-Br-2-Cl-phenol, NaI, Cs_2_CO_3_, DMF, 80 °C, 10 h; (**e**) but-3-yn-ol, Pd(PPh_3_)_4_, CuI, NEt_3_, DMSO, r.t., 16 h; (**f**) PtO_2_, H_2_, MeOH, r.t, 16 h; (**g**) PDC, DMA, r.t., 16 h; (**h**) (i) VHL-1, DMA, HATU, DIPEA, r.t., 16 h; (ii) 4M HCl in dioxane, THF, r.t., 2 h.

**Scheme 17:**
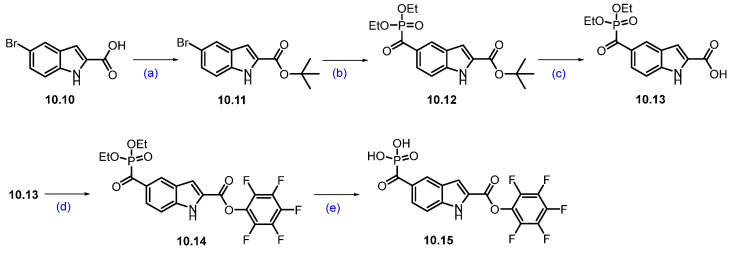
Synthesis of fragment **10.15** of **KT-333 (10):** (**a**) *tert*-butyl 2,2,2-trichloroacetimide, BF_3_·Et_2_O, THF, 0 °C to r.t., 16 h; (**b**) CO, Pd_2_(dba)_3_, Xantphos, diethylphosphonate, NEt_3_, toluene, 90 °C, 4 h; (**c**) TFA, DCM, r.t., 2 h; (**d**) 2,3,4,5,6-pentafluorophenol, DCC, DCM, r.t., 16 h; (**e**) TMSI, DCM, r.t., 30 min.

**Scheme 18:**
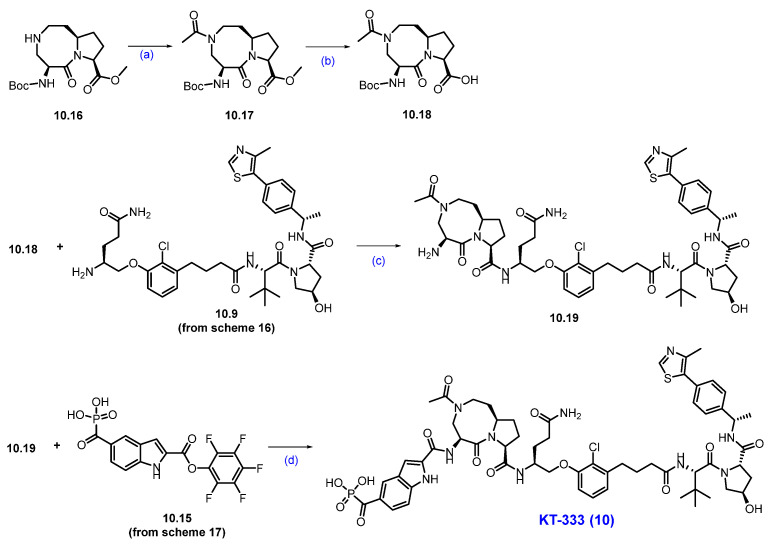
Synthesis of **KT-333 (10):** (**a**) AcCl, NEt_3_, DCM, 0 °C to r.t., 3 h; (**b**) LiOH, THF/H_2_O, r.t, 16 h; (**c**) HATU, DIPEA, DMA, 0 °C to r.t., 16 h; HCl in dioxane, DCM, r.t., 5 h; (**d**) DIPEA, NMP, ACN, r.t.

Synthesis of **KT-333 (10):** The synthesis of KT-333 is shown in Scheme 16, Scheme 17 and Scheme 18 [95], beginning with the commercially available starting material **10.1**. Methyl (*tert*-butoxycarbonyl)-*L*-glutaminate **10.2** was prepared via esterification of (*tert*-butoxycarbonyl)-*L*-glutamine **10.1**. The ester group in compound **10.2** was then reduced to the corresponding alcohol **10.3** using NaBH_4_ in THF. The resultant alcohol was subsequently converted to mesylate **10.4** by treatment with mesyl chloride and triethylamine.

Next, a substitution reaction between mesylate **10.4** and 3-bromo-2-chlorophenol in the presence of Cs_2_CO_3_ afforded phenol ether **10.5**. A chemo-selective Sonogashira coupling of the bromide of **10.5** with but-3-yn-1-ol furnished alkyne derivative **10.6**. This alkyne was then subjected to hydrogenation using platinum oxide as a catalyst to yield the corresponding saturated alcohol **10.7**.

Subsequent oxidation of compound **10.7** with pyridinium dichromate (PDC) produced carboxylic acid **10.8**. Further compound **10.8** was coupled with the VHL ligand using HATU and DIPEA in dimethylacetamide (DMA) to form a Boc-protected coupled product. Finally, the Boc deprotection was carried out using HCl in dioxane/THF mixture at room temperature, furnished the target compound **10.9** (Scheme 16) [96].

The synthesis of compound **10.15** was started with commercially available bromo acid **10.10**. *tert*-Butyl-5-bromo-1*H*-indole-2-carboxylate **10.11** was synthesized by esterifying commercially available starting material 5-bromo-1*H*-indole-2-carboxylic acid **10.10** with *tert*-butyl-2,2,2-trichloroacetimidate and BF_3_-etherate in THF. The bromo indole derivative **10.11** was then converted into a phosphate ester **10.12** using a palladium-catalyzed coupling with diethyl phosphonate in the presence of carbon monoxide. The *tert*-butyl group in compound **10.12** was then deprotected using TFA in DCM to furnish **10.13**. The indole carboxylic acid derivative **10.13** was then coupled with pentafluorophenol using DCC and DMAP in DCM to afford compound **10.14**. Phosphate ester hydrolysis of **10.14** using TMSI in DCM produced compound **10.15** in 78% yield (Scheme 17) [97].

The final synthesis of KT-333 is described in Scheme 18. The synthesis started with compound **10.16**, which was converted to the corresponding acetamide **10.17** using acetyl chloride and triethylamine. Subsequently, **10.17** was subjected to ester hydrolysis with LiOH in THF and water to afford acid **10.18**. The acid **10.18** was then coupled with previously synthesized amine derivative **10.9** (Scheme 16) using HATU and DIPEA to produce a Boc-protected compound, which was next subjected to Boc deprotection with HCl to afford amine **10.19**. In the final step, compounds **10.19** and **10.15** (Scheme 17) were coupled in the presence of DIPEA in NMP to furnish **KT-333 (10)** in good yield [96].

### 2.7. PROTACs Targeting B Cell Lymphoma 6 (BCL6)

Diffuse large B-cell lymphoma (DLBCL) is the most common type of lymphoma, originating from the uncontrolled proliferation of B cells. It accounts for approximately 25–30% of all non-Hodgkin’s lymphomas. This disease is highly heterogeneous, with 20–40% of patients exhibiting mutations in the B cell lymphoma 6 (BCL6) gene [98]. BCL6 plays a crucial role as a transcription factor, essential for regulating cell cycle checkpoints, differentiation, apoptosis, and the DNA damage response. Evidence has increasingly suggested that sustained expression of BCL6 is necessary for the survival of DLBCL cells and is associated with the occurrence of various cancers [99]. Over the past few decades, significant efforts have been directed toward developing BCL6 inhibitors to interrupt the protein–protein interactions between the BCL6 BTB domain and its corepressor. While some compounds have effectively blocked these interactions, most inhibitors have demonstrated suboptimal antiproliferative effects, and currently, none have entered clinical development [100,101,102].

Notably, Arvinas and Bristol Myers Squibb have independently announced two CRBN-based BCL6-targeting PROTACs, ARV-393 and BMS-986458, which have progressed into early clinical studies. ARV-393 is being tested in patients with relapsed/refractory non-Hodgkin lymphoma (NCT06393738). Meanwhile, BMS-986458 is undergoing Phase 1/2 clinical trials for the same condition, being evaluated as a standalone treatment and in combination with the monoclonal antibody rituximab (NCT06090539) [103].

Clinical Development and Outcomes of **ARV-393 (11):** Arvinas presented new preclinical combination data for ARV-393, their investigational PROTAC BCL6 degrader, at the 2025 AACR Annual Meeting [104]. The data showed synergistic antitumor activity, including complete regressions, when combined with standard-of-care (SOC) chemotherapy, SOC biologics, and investigational oral small-molecule inhibitors (SMIs) in models of high-grade B-cell lymphoma (HGBCL) and aggressive diffuse large B-cell lymphoma (DLBCL). The combination of ARV-393 and SOC chemotherapy (rituximab, cyclophosphamide, doxorubicin, vincristine, and prednisone) achieved significantly greater tumor growth inhibition compared to ARV-393 monotherapy, with all mice receiving the ARV-393 and R-CHOP combination exhibiting complete tumor regression. Furthermore, ARV-393 combined with SOC biologics targeting CD20 (rituximab), CD19 (tafasitamab), or CD79b (polatuzumab vedotin) resulted in tumor regressions and demonstrated significantly more potent tumor growth inhibition than each agent used alone. In preclinical models, ARV-393 also increased CD20 expression, supporting further exploration of combinations with CD20-targeted agents, particularly in cases of low or absent CD20 expression. Moreover, when combined with investigational small-molecule inhibitors targeting clinically validated oncogenic drivers of lymphoma, such as BTK (Acalabrutinib), Bcl2 (Venetoclax), or EZH2 (Tazemetostat), ARV-393 demonstrated superior tumor growth inhibition compared to each agent alone. All mice treated with these combinations showed tumor regressions [104]. These preclinical data suggest that ARV-393 holds promise as a novel therapeutic approach for lymphoma patients, particularly those who have not responded to existing treatments.

**Scheme 19:**
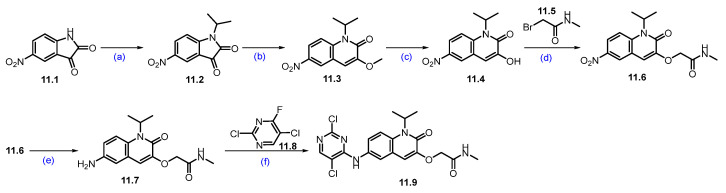
Synthesis of fragment **11.9** of **ARV-393 (11):** (**a**) 2-iodopropane, K_2_CO_3_, DMF, r.t., 48 h; (**b**) TMSCH_2_N_2_, NEt_3_, EtOH, r.t., 12 h; (**c**) BBr_3_, DCM, 0 °C, 2 h; (**d**) K_2_CO_3_, DMF, 20 °C, 4 h; (**e**) 10%Pd/C, H_2_, MeOH/DMF, r.t., 4 h; (**f**) DIPEA, DMSO, 100 °C, 2 h.

**Scheme 20:**
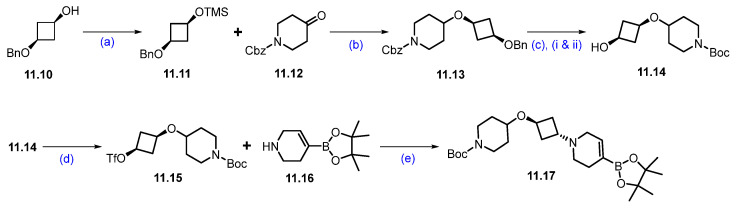
Synthesis of fragment **11.17** of **ARV-393 (11):** (**a**) TMSCl, NEt_3_, DCM, 0 °C to 2 h; (**b**) triethylsilane, DCM, TMS triflate, −78 °C to 0 °C, 2 h; (**c**) (i) 10%Pd/C, Pd(OH)_2_/C, 15 psi, 55 °C, H_2_, 48 h; (ii) (Boc)_2_O; (**d**) triflic anhydride, NEt_3_, −40 °C, 2 h; (**e**) DIPEA, ACN, r.t., 5 h.

**Scheme 21:**
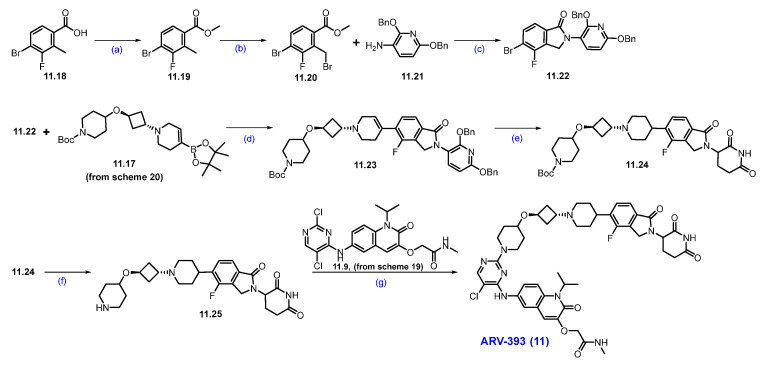
Synthesis of **ARV-393 (11):** (**a**) H_2_SO_4_, MeOH, 0 °C to 60 °C 16 h; (**b**) NBS, AIBN, 1,2-DCE, 80 °C, 6 h; (**c**) DIPEA, ACN, 70 °C, 16h; (**d**) Pd(dppf)Cl_2_, CsF, dioxane/H_2_O, 90 °C; (**e**) 10%Pd/C, H_2_, EtOH, r.t., 16 h; (**f**) HCl in dioxane, r.t., 2 h; (**g**) DIPEA, DMSO, 120 °C, 1 h.

Synthesis of **ARV-393 (11)**: The synthesis of **ARV-393** is shown in Scheme 19, Scheme 20 and Scheme 21 [105]. The synthesis of ARV-393 requires three key building blocks and is completed in a total of 18 steps.

The reaction commenced with the *N*-alkylation of 5-nitro-indoline-2,3-dione **11.1** with 2-iodopropane using potassium carbonate (K_2_CO_3_), which afforded alkylated product **11.2**. The ring expansion reaction of **11.2** with TMS-diazomethane and triethylamine (NEt_3_) obtained quinoline derivative **11.3**, which was further treated with boron tribromide (BBr_3_) to obtain hydroxy derivative **11.4**. It was subjected to *O*-alkylation with K_2_CO_3_ to furnish the ether derivative **11.6**. The nitro group reduction of **11.6** was carried out with Pd/C under hydrogenation conditions, which gave amino quinoline **11.7**. S_N_Ar reaction of amine on **11.7** with **11.8** using DIPEA at elevated temperature yielded regiomeric amine substituted product **11.9** (Scheme 19) [106].

The synthesis of the second fragment begins with the trimethylsilyl protection of commercially available cis-3-(benzyloxy)cyclobutane-1-ol, resulting in the formation of the di-protected cyclobutyl derivative **11.11**. This scaffold is then subjected to Lewis acid-catalyzed etherification with triethylsilane, which yields the ether derivative **11.13**. Subsequently, hydrogenation of compound **11.13** with Pd/C, followed by in situ treatment with Boc anhydride, facilitates the substitution of the -Cbz protection group with Boc protection, resulting in compound **11.14**. This compound is then treated with trifluoromethanesulfonic anhydride in the presence of triethylamine to produce the reactive triflate **11.15**. Finally, **11.15** was reacted with commercially available compound **11.16**, which afforded *N*-alkylated derivative **11.17** (Scheme 20) [105].

Esterification of 4-bromo-3-fluoro-2-methylbenzoic acid **11.18** was carried out using MeOH and H_2_SO_4_ to obtain ester **11.19**, which was further treated with NBS in the presence of a catalytic amount of AIBN in CCl_4_, affording benzyl bromide derivative **11.20**. Condensation of **11.20** with 2,6-bis(benzyloxy)pyridine-3-amine using DIPEA and a catalytic amount of acetic acid in acetonitrile obtained substituted isoindolone derivative **11.22**. Suzuki coupling reaction of **11.22** with **11.17** using Pd(dppf)Cl_2_, CsF in dioxane at 90 °C rendered coupling product **11.23**. Furthermore, it was subjected to hydrogenation to remove dibenzyl functionalities, followed by Boc group deprotection using HCl in dioxane, which furnished the key scaffold **11.25**. Finally, the nucleophilic substitution of the secondary amine of **11.25** on 2-Cl-pyrimidine of **11.9** (Scheme 19) resulted in **ARV-393 (11)** (Scheme 21) [106].

**Scheme 22:**
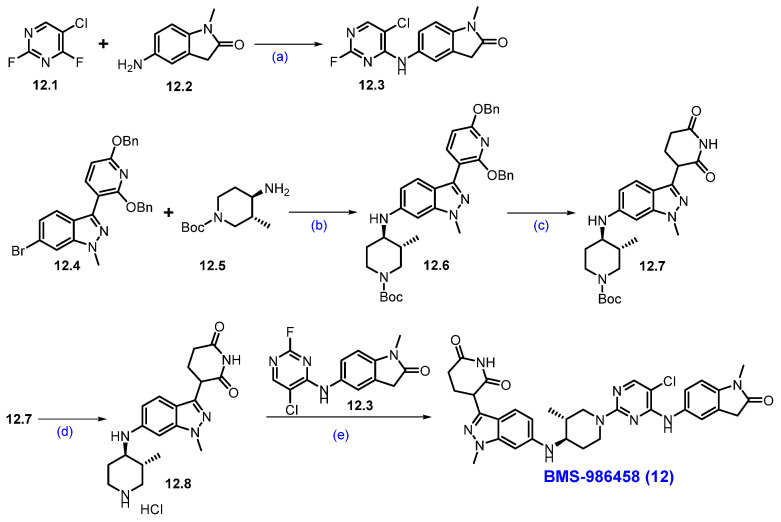
Synthesis of **BMS-986458** (**12**): (**a**) DIPEA, THF, −40 °C to r.t., 16 h; (**b**) Ruphos-Pd-G3, Cs_2_CO_3_, 1,4-dioxane, 90 °C, 20 h; (**c**) Pd(OH)_2_/C, H_2_, MeOH, THF, 50 °C, 9 h; (**d**) 4M HCl in EtOAc, r.t., 16 h; (**e**) DIPEA, DMSO, 80 °C, 2 h.

Synthesis of **BMS-986458 (12)**: The synthesis of BMS-986458 is described in Scheme 22 [107]. The synthesis began with commercially available 5-chloro-2,4-difluoropyrimidine **12.1** and 5-amino-l-methylindolin-2-one **12.2**. These two compounds reacted in THF in the presence of DIPEA at a lower temperature, yielding product **12.3**. For the synthesis of cereblon ligand part, the synthesis began utilizing a literature-reported compound **12.4** [108], which was converted to intermediate **12.6** by following a Buchwald-Hartwig reaction with amine **12.5** in the presence of Ruphos-Pd-G3 and Cs_2_CO_3_ in 1,4-dioxane. Then, compound **12.6** was treated with Pd(OH)_2_/C under a hydrogen atmosphere to deprotect the benzyl protecting groups, which gave piperidine-2,6-dione derivative **12.7**. Further deprotection of the Boc functionality on **12.7** in the presence of 4M HCl in EtOAc furnished amine **12.8**. Finally, a S_N_Ar reaction between amine **12.8** and **12.3** in DMSO, in the presence of DIPEA, produced the final degrader **BMS-986458** (**12**).

### 2.8. PROTACs Targeting Murine Double Minute 2 (MDM2)

Addressing elevated protein levels resulting from off-target effects remains a significant challenge, particularly when targeting MDM2. A primary concern is the inadvertent activation of p53, a vital tumor suppressor, in healthy tissues. The MDM2 protein serves as a key cellular regulator of the tumor suppressor p53, often referred to as the “guardian of the genome.” p53 is essential for cancer prevention through its control of DNA repair, cell cycle progression, apoptosis, angiogenesis, and senescence [109]. Notably, mutations in the *TP53* gene are found in approximately 50% of all human cancers, including those of the breast, colon, lung, liver, prostate, bladder, and skin [110]. As an E3 ligase, MDM2 targets p53 for degradation, making it an essential focus for cancer treatments. However, efforts to stabilize and enhance p53 expression using small molecules face challenges due to a negative feedback loop in which upregulated p53 increases the transcription of MDM2 messenger RNA. More than a dozen potent inhibitors designed to disrupt the MDM2–p53 interaction have entered clinical trials, but these agents often face setbacks [111,112]. Key challenges include dose-limiting toxicities (DLTs) from p53 activation in bone marrow, a narrow therapeutic window because of p53′s importance in normal cells, and resistance mechanisms involving *TP53* mutations. Kymera Therapeutics has recently introduced KT-253, an MDM2 PROTAC degrader (NCT05775406). Unlike conventional small-molecule inhibitors, which may unintentionally upregulate MDM2 through feedback, KT-253 rapidly depletes MDM2 and triggers a sharp increase in p53 levels. This mechanism could support more robust and rapid therapeutic responses, enable intermittent dosing, mitigate toxicity, and give healthy tissues time to recover.

KT-253 was administered intravenously every three weeks to patients with advanced high-grade myeloid malignancies, such as acute myeloid leukemia (AML), acute lymphoblastic leukemia, lymphomas, and various solid tumors. Thus far, no treatment-related neutropenia or thrombocytopenia has been observed, and early signs of therapeutic efficacy have been noted at tolerated doses. However, on 31 May 2025, Kymera announced it would shift its focus and resources away from oncology to prioritize its growing immunology portfolio. As a result, further development of KT-253 beyond Phase 1 will proceed only through external partnerships [113]. The use of a PROTAC to target MDM2 illustrates an innovative strategy that employs one E3 ligase to degrade another E3 ligase.

Clinical Development and Outcomes of **KT-253 (13):** As of April 2024, an ongoing Phase 1a clinical trial has enrolled 24 patients: 16 with solid tumors and lymphomas (Arm A) and 8 with high-grade myeloid malignancies (Arm B) [114,115,116]. Preliminary data show encouraging activity. Arm A reported one confirmed partial response in a patient with Merkel cell carcinoma, along with four cases of stable disease in individuals with fibromyxoid sarcoma, adenoid cystic carcinoma, and renal cell carcinoma. Arm B showed one confirmed complete response and one partial response in post-myeloproliferative neoplasm acute myeloid leukemia patients. Treatment was associated with activation of the p53 pathway, demonstrated by elevated plasma levels of GDF-15 protein and increased expression of CDKN1A and PHLDA3 mRNA, even at the lowest dose levels, confirming successful MDM2 inhibition. KT-253 was generally well-tolerated; no cases of neutropenia or thrombocytopenia were observed. These early findings support KT-253′s potential to effectively inhibit MDM2, restore p53 function, and drive tumor regression in patients with p53 wild-type cancers [117].

**Scheme 23:**
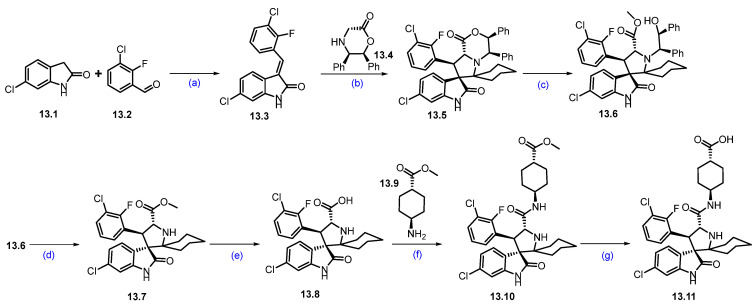
Synthesis of fragment **13.11** of **KT-253 (13):** (**a**) Piperidine, MeOH, 65 °C for 5 h then at r.t. for 12 h; (**b**) toluene, cyclohexanone, 140 °C, 12 h; (**c**) H_2_SO_4_, MeOH, 50 °C, 5 h; (**d**) CAN, ACN/H_2_O, r.t., 30 min.; (**e**) LiOH, THF/H_2_O, r.t., 15 min.; (**f**) TCFH, NMI, 0 °C to r.t., 2 min.; (**g**) LiOH, THF/H_2_O, r.t., 30 min.

**Scheme 24:**
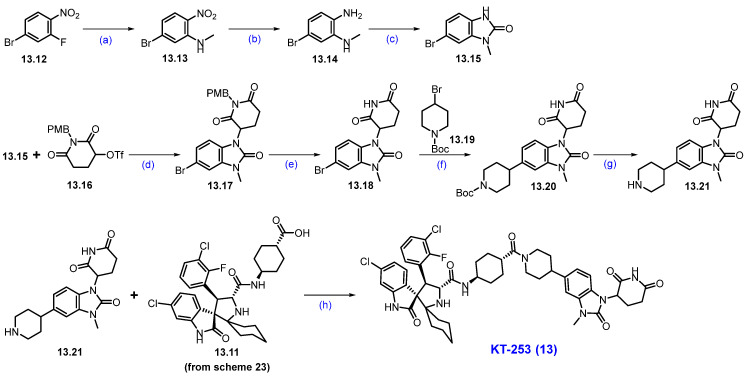
Synthesis of **KT-253 (13):** (**a**) MeNH_2_, THF, 15 °C, 10 min.; (**b**) Fe, AcOH, EtOAC, 80 °C, 6 h; (**c**) CDI, ACN, 80 °C, 6 h; (**d**) *t*-BuOK, THF, 0 °C to 10 °C, 2.5 h; (**e**) CH_3_SO_3_H, toluene, 120 °C, 2 h; (**f**) Ni/Ir photocatalysis, 50 W blue LED lamp, r.t., 14 h; (**g**) TFA, DCM, r.t., 30 min.; (**h**) TCFH, NMI, ACN, r.t., 2 min.

Synthesis of **KT-253 (13**): The synthesis of **KT-253** is summarized in Scheme 23 and Scheme 24 [118]. Condensation of 3-chloro-2-fluoro benzaldehyde **13.2** with 6-chloroindolin-2-one **13.1** under basic conditions using piperidine in MeOH at reflux conditions afforded **13.3** as the exclusive desired *E*-isomer. Next, the asymmetric synthesis of the spirooxoindole core was achieved using **13.4** as a chiral auxiliary, with cyclohexanone at 140 °C, resulting in the cycloaddition product **13.5**. It was treated with H_2_SO_4_ in MeOH, resulting in ring opening followed by in situ acid-catalyzed esterification product **13.6**. Oxidative removal of the chiral auxiliary by ceric ammonium nitrate (CAN) rendered **13.7**. Hydrolysis of **13.7** using aq. LiOH followed by amidation with trans cyclohexyl carboxylate **13.9** afforded **13.10**. Next, ester hydrolysis of **13.10** with aq. LiOH resulted in key spirooxoindole scaffold **13.11** (Scheme 23).

In parallel, substituted benzimidazole moiety **13.21** was synthesized in 7 steps (Scheme 24). An S_N_Ar reaction of **13.12** with methylamine afforded 5-bromo-*p*-nitro-*N*-methylaniline derivative **13.13**, which was subjected to reduction in the nitro moiety with Fe/AcOH, affording 1,2-diamino-bromobenzene **13.14**. The cyclization of **13.14** with 1,1′-carbonyldiimidazole (CDI) at elevated temperature furnished benzimidazolone scaffold **13.15**. Condensation of benzimidazole derivative **13.15** with **13.16** using potassium *tert*-butoxide (*^t^*BuOK) in THF conditions resulted in the formation of *N*-alkylated product **13.17**. It was further treated with methanesulfonic acid (CH_3_SO_3_H) in toluene at 120 °C, resulting in **13.18**. The compound then underwent a Ni/Ir photocatalyzed coupling between aryl bromide **13.18** and alkyl bromide **13.19** under irradiation using a 50W blue LED lamp, which afforded the newly formed C-C bond coupled product **13.20** in high yields. Finally, the deprotection of the Boc group in **13.20**, using TFA, yielded **13.21**. The resultant piperidine amine **13.21** was used in a coupling reaction with a previously synthesized carboxylic acid **13.11** (Scheme 23) under HATU and DIPEA conditions, furnishing **KT-253 (13)**.

### 2.9. PROTACs Targeting Switch/Sucrose Non-Fermentable Related, Matrix Associated, Actin Dependent Regulator of Chromatin, Subfamily A, Member 2 (SMARCA2)

SMARCA2 and SMARCA4 are mutually exclusive, catalytically active components of the BAF chromatin remodeling complex and exhibit a synthetic lethal relationship [119]. Approximately 20% of human cancers harbor mutations in BAF complex subunits, with loss-of-function alterations in SMARCA4 particularly prevalent in malignancies such as non-small cell lung cancer (NSCLC). SMARCA2 and SMARCA4 share a high degree of sequence homology, including a conserved ATPase domain (93% similarity) and a bromodomain (96% similarity), both of which recognize acetylated chromatin [120]. Although small-molecule inhibitors have been developed to target the ATPase and bromodomains of SMARCA2/4, their high sequence similarity poses a significant challenge for achieving target selectivity. Most of these inhibitors fail to distinguish between SMARCA2 and SMARCA4. Moreover, bromodomain inhibition does not appear to affect cell viability in tumor models, suggesting that the bromodomain is not essential for cancer cell survival. As a result, a more promising strategy is the selective degradation of SMARCA2 in cancers where SMARCA4 is genetically inactivated [121,122,123].

PROTACs offer a unique advantage in this context. Despite originating from ligands that bind both SMARCA paralogs, PROTACs have demonstrated the ability to selectively degrade SMARCA2 while sparing SMARCA4. This selective degradation is especially compelling given the structural and functional similarities between the two proteins [124,125,126,127,128].

Prelude Therapeutics is developing two SMARCA2-selective PROTACs currently in Phase I clinical trials for patients with SMARCA4-deficient solid tumors: PRT3789, an intravenous agent (NCT05639751), and PRT7732, an oral compound with an undisclosed structure (NCT06560645). PRT3789 was specifically engineered with high aqueous solubility and an extended in vivo half-life—attributes well-suited for VHL-based heterobifunctional degraders [129,130]. A recent study by Shcherbakov et al. provides valuable insight into the basis of SMARCA2 selectivity through comprehensive biophysical characterization [131]. Using a VHL-recruiting PROTAC, the researchers examined ternary complex formation with both SMARCA2 and SMARCA4. HAP-NMR analysis revealed that the SMARCA2-containing ternary complex exhibited greater cooperativity and slower molecular tumbling in solution. Furthermore, isothermal titration calorimetry (ITC) using micelle-solubilized PROTACs demonstrated that SMARCA2 complexes were more enthalpically favorable and entropically efficient. These results are further corroborated by a related study from Wurz et al., which discovered that a single amino acid difference between SMARCA2 and SMARCA4 affects structural cooperativity during the formation of the ternary complex [132].

**Scheme 25:**
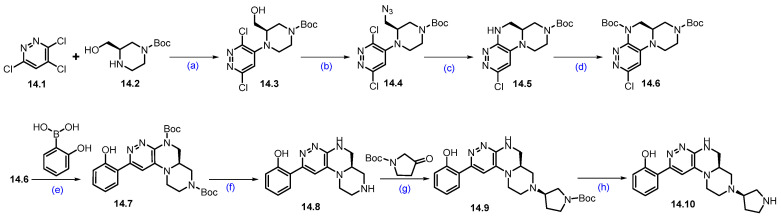
Synthesis of fragment **14.10** of **PRT3789 (14):** (**a**) DIPEA, DMF, 80 °C, 16 h; (**b**) TPP, DIAD, DPPA, THF, 0 °C to r.t., 16 h; (**c**) TPP, THF, 60 °C, 3 h then H_2_O, DIPEA, 60 °C, 20 h; (**d**) (Boc)_2_O, DMAP, DCM, r.t., 1 h; (**e**) Pd(dppf)Cl_2_.DCM, K_2_CO_3_, dioxane; 105 °C, 18 h; (**f**) TFA, DCM, r.t., 1 h; (**g**) HCl, MeOH, r.t., 1 h; (**h**) TFA, DCM, r.t., 16 h.

**Scheme 26:**
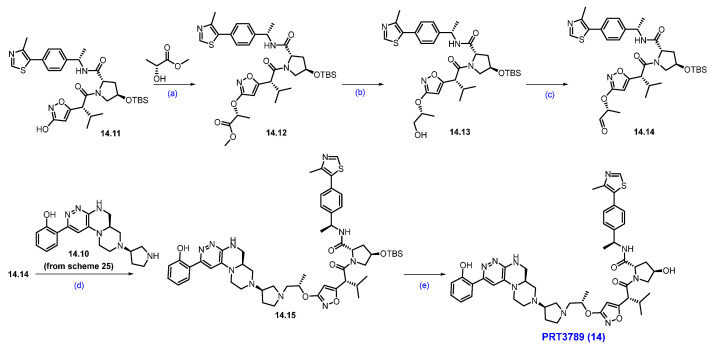
Synthesis of **PRT3789 (14):** (**a**) TPP, DIAD, THF, 0 °C to r.t., 16 h; (**b**) NaBH_4_, MeOH, r.t., 3 h; (**c**) IBX, ACN, 80 °C, 3 h; (**d**) Na(OAc)_3_BH, NaHCO_3_, DCM, rt.,18 h; (**e**) TFA, DCM, r.t., 1 h.

Synthesis of **PRT3789 (14):** The synthesis of PRT3789 is summarized in Scheme 25 and Scheme 26 [133]. Synthesis initiated with the S_N_Ar reaction of commercially available 3,4,6-trichloropyridazine **14.1** and (*R*)-1-Boc-3-hydroxymethylpiperazine **14.2** using DIEPA in DMF afforded regiomeric piperazine-substituted scaffold **14.3**. Conversion of hydroxymethyl on **14.3** under Mitsunobu conditions using diphenyl phosphoryl azide (DPPA) resulted in azidomethyl azide compound **14.4**. Further reduction of azide with triphenylphosphine in THF, followed by cyclization with DIPEA, afforded cyclized scaffold **14.5**. The latter was treated (TPP) with Boc anhydride in the presence of catalytic DMAP to yield Boc-protected scaffold **14.6**. It was subjected to a Suzuki coupling reaction using 2-hydroxyphenylboronic acid in the presence of Pd(dppf)Cl_2_.DCM, K_2_CO_3_ conditions to give product **14.7**. Deprotection of di-Boc functionalities in **14.7** was carried out with TFA to yield **14.8**. Reductive amination of **14.8** with commercially available *tert*-butyl 3-oxopyrrolidine-1-carboxylate using Na(OAc)_3_BH followed by SFC purification and Boc deprotection under HCl/MeOH conditions to obtain key scaffold **14.10** (Scheme 25).

The Mitsunobu coupling reaction of **14.11** with commercially available methyl-(*R*)-2-hydroxypropanoate under TPP/DIAD conditions afforded scaffold **14.12** as shown in Scheme 26. It was subjected to ester reduction with NaBH_4_ in MeOH, followed by oxidation with IBX, which obtained the key aldehyde **14.14**. Finally, the reductive amination of **14.10** (Scheme 25) with aldehyde **14.14** yielded the reductive amination scaffold **14.15**. The silyl deprotection under acidic conditions furnished **PRT3789 (14)** [134].

### 2.10. PROTACs Targeting B-Raf Proto-Oncogene, Serine/Threonine Kinase (BRAF)

The RAS-RAF-MEK-ERK signaling cascade plays a critical role in maintaining cellular homeostasis. Class 1 BRAF mutations, such as V600E and V600K, lead to hyperactivation of this pathway, driving uncontrolled cell growth. BRAF mutations are found in approximately 8% of all cancers, with high prevalence in melanoma (60%), colorectal cancer (10%), non-small cell lung cancer (NSCLC, 10%), and hairy cell leukemia (100%) [135]. RAF inhibitors like vemurafenib have improved PFS in patients harboring the BRAF V600E mutation. However, resistance to these inhibitors often emerges, along with paradoxical activation of RAF. Although some kinase inhibitors in clinical development aim to counter this effect, targeted protein degraders offer a potentially more effective strategy by eliminating both normal and mutant RAF activity [136]. CFT1946 is an oral, CRBN-based PROTAC developed by C4 Therapeutics to target and degrade mutant BRAF V600E. It is currently being investigated in a Phase 1/2 clinical trial (NCT05668585), both as a monotherapy and in combination with the MEK1/2 inhibitor trametinib, for patients with BRAF V600-driven cancers, including those resistant to standard BRAF inhibitors [137].

Clinical Development and Outcomes of **CFT1946 (15):** As of 12 April 2024, early results from an ongoing Phase 1/2 clinical trial of CFT1946 have been released. In the monotherapy dose-escalation phase, 25 patients with BRAF V600-mutant solid tumors were treated with CFT1946 at doses ranging from 20 mg to 320 mg twice daily. The patient population consisted of individuals with melanoma (40%), colorectal cancer (40%), non-small cell lung cancer (8%), and other tumor types (12%). CFT1946 exhibited dose-dependent bioavailability and effectively degraded the BRAF V600E protein. The treatment was generally well-tolerated, with most adverse events reported as mild to moderate. Importantly, signs of anti-tumor activity were observed, reinforcing the therapeutic potential of CFT1946 for patients with BRAF V600-mutant solid tumors [138,139].

**Scheme 27:**
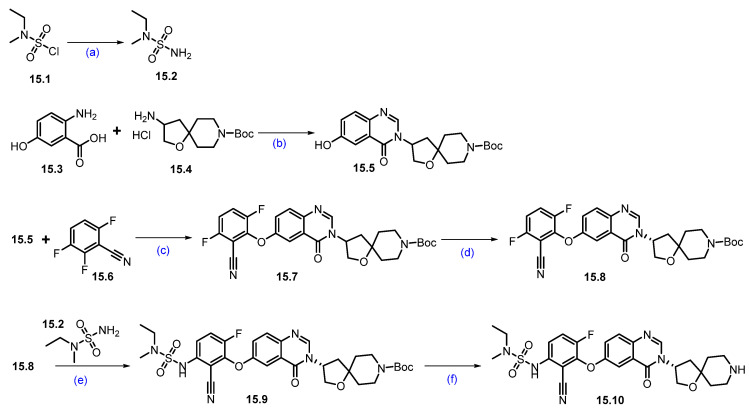
Synthesis of fragment **15.10** of **CFT1946 (15):** (**a**) 7N NH_3_ in MeOH, −10 °C to r.t., 14 h; (**b**) CH(OEt)_3_, toluene, THF, 110 °C, sealed tube, 18 h; (**c**) KO^t^Bu, DMF/THF mixture, r.t., 14 h; (**d**) Chiral SFC; (**e**) Cs_2_CO_3_, DMF, 65 °C, 16 h; (**f**) 4N HCl in dioxane, r.t., 4 h.

**Scheme 28:**
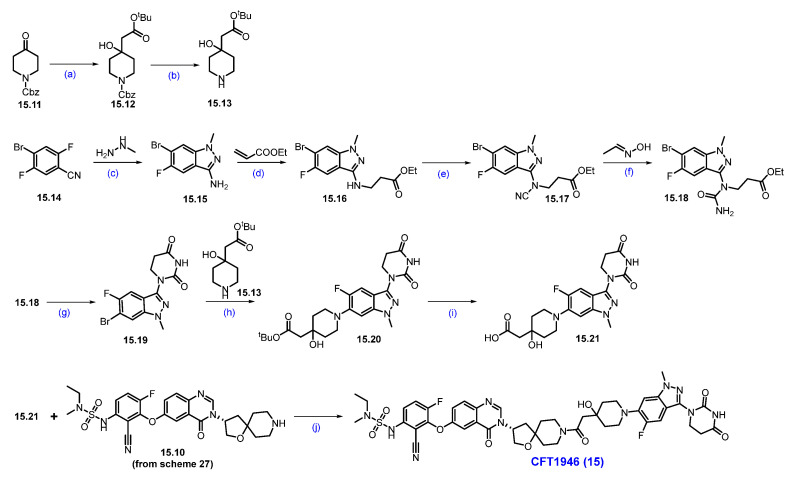
Synthesis of **CFT1946 (15):** (**a**) LDA, THF, −78 °C for 1 h then −78 °C to −10 °C for 2 h; (**b**) H_2_, 10% Pd/C, 1,4-dioxane; (**c**) EtOH, 80 °C, 12 h; (**d**) DBU-Lac, ionic liquid, 90 °C, 48 h; (**e**) CNBr, NaOAc, EtOH, 80 °C, 16 h; (**f**) InCl_3_ (cat.), toluene, reflux, 1 h; (**g**) triton-B, acetonitrile, r.t., 1 h; (**h**) Pd-PEPPSI-iHept(Cl), Cs_2_CO_3_, 1,4-dioxane, 110 °C, 2 h; (**i**) 4N HCl in dioxane, DCM, r.t., 6 h; (**j**) HATU, DIPEA, DMF, r.t., 12 h.

Synthesis of **CFT1946 (15):** In Scheme 27 and Scheme 28, the synthesis of CFT1946 is presented as described by C4 therapeutics in their patents [140]. The quinazolinone scaffold **15.10** was synthesized starting from the 2-amino-5-hydroxy benzoic acid **15.3**, as shown in Scheme 27. Briefly, a dehydrative coupling reaction of **15.3** and **15.4** in the presence of triethyl orthoformate resulted in quinazolinone framework **15.5**. Aromatic nucleophilic substitution on **15.6** with the phenoxide generated from **15.5** afforded compound **15.7,** furthering chiral purification, and resulting in the desired enantiopure nitrile **15.8**. A second aromatic nucleophilic substitution of **15.8** with **15.2** furnished **15.9**, which on Boc deprotection resulted in amine **15.10**.

The nucleophilic addition of the carbanion generated from *tert*-butyl acetate to the carbonyl group of **15.11** generated alcohol **15.12**, which, on Cbz deprotection with Pd/C and H_2_, furnished amine **15.13** as shown in Scheme 28. The indazole framework **15.15** was synthesized from substituted benzonitrile **15.14** and methyl hydrazine. Michael addition reaction of amine in **15.15** with ethyl acrylate in the presence of [DBU]·[Lac] ionic liquid furnished **15.16**. *N*-cyanation of **15.16** with cyanogen bromide (CNBr) and NaOAc produced **15.17**. An acid-catalyzed hydrolysis of the nitrile group followed by a cyclization reaction in the presence of Triton-B furnished compound **15.19**. Subsequent Buchwald Hartwig coupling reaction of **15.19** with amine **15.13** furnished ester **15.20**, which was converted to acid **15.21** by the deprotection of the *tert*-butyl group with HCl in dioxane. A final HATU-mediated acid–amine coupling reaction with amine **15.10** and previously prepared acid **15.21** (Scheme 27) resulted in **CFT1946 (15)**.

### 2.11. PROTACs Targeting Kirsten Rat Sarcoma Virus (KRAS)

KRAS is a critical regulator of the MAPK signaling pathway, which governs essential cellular processes such as proliferation, migration, and gene expression. Activating mutations in KRAS are commonly found in several cancers, most notably pancreatic adenocarcinoma, non-small cell lung cancer (NSCLC), and colorectal cancer. These mutations result in constitutive activation of the MAPK pathway, driving uncontrolled tumor growth.

Historically, KRAS was deemed “undruggable” due to its strong binding affinity for GDP/GTP and the lack of suitable hydrophobic pockets for small-molecule targeting [141,142,143]. This perception shifted with the discovery of sotorasib and adagrasib, the first FDA-approved KRAS inhibitors [144,145,146]. These agents target the inducible switch II pocket and covalently bind to KRAS-GDP by exploiting the reactive cysteine residue introduced by the G12C mutation.

Expanding on this breakthrough, Mirati Therapeutics developed MRTX1133, the first selective and potent reversible inhibitor of KRASG12D, which was derived from an earlier irreversible G12C inhibitor [147]. While its entry into Phase 1 clinical trials in 2023 marked significant progress, the rapid emergence of resistance previously observed with KRAS G12C inhibitors remains a major concern [148]. Such resistance may arise from secondary mutations in KRAS, gene amplification, or compensatory reactivation of the MAPK pathway.

To address these limitations, targeted degradation of KRASG12D offers a promising alternative. ASP3082, developed by Astellas Pharma, is a KRASG12D selective degrader that employs VHL-mediated E3 ligase recruitment to eliminate the mutant protein [149,150]. Currently in Phase 1 clinical development, ASP3082 was granted FDA fast track designation in February 2023 for the treatment of pancreatic adenocarcinoma (NCT05382559).

**Scheme 29:**
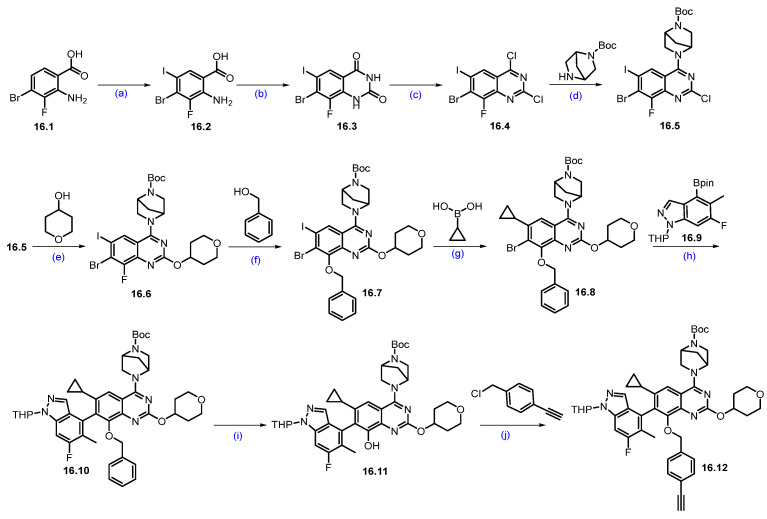
Synthesis of fragment **16.11** of **ASP3082 (16):** (**a**) NIS, DMF, 50 °C, 4 h; (**b**) Urea, 200 °C, 3 h; (**c**) POCl_3_, DIPEA, reflux, 16 h; (**d**) DIPEA, THF, 0 °C, 16 h; (**e**) Cs_2_CO_3_, DABCO, DMF, THF, r.t., 16 h; (**f**) *t*-BuOH, THF, 0 °C, 1.5 h; (**g**) Pd(dppf)Cl_2_, K_3_PO_4_, ACN, 1,4-dioxane, H_2_O, 100 °C, 3 h; (**h**) RuPhos PdG3, RuPhos, K_3_PO_4_, 1,4-dioxane, 100 °C, 2.5 h; (**i**) 10% Pd/C/H_2_, r.t., 2 h; (**j**) Cs_2_CO_3_, THF, 60 °C, 2 h.

**Scheme 30:**
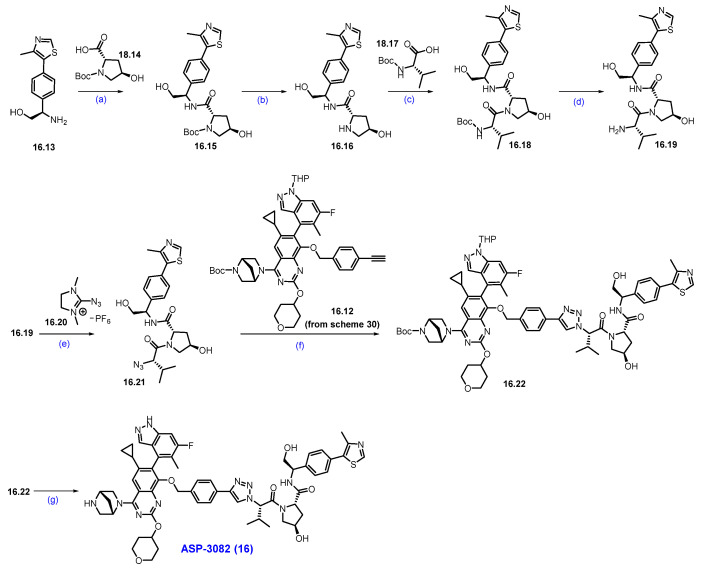
Synthesis of **ASP-3082 (16):** (**a**) HATU, DIPEA, DMF, 5 °C for 1 h then at r.t., for 1 h; (**b**) HCl in dioxane, MeOH/DCM mixture, 0 °C to r.t., 6 h; (**c**) HATU, DIPEA, DMF, 5 °C for 1 h then at r.t., for 1h; (**d**) HCl in dioxane, 0 °C to r.t., 5 h; (**e**) NEt_3_, ACN, THF, 0 °C, 5 h; (**f**) Sodium ascorbate, CuI, *t*-BuOH, THF, H_2_O, 50 °C, 3 h; (**g**) TFA, DCM, 0 °C to r.t., 2 h.

Synthesis of **ASP3082 (16):** The synthesis of ASP-3082 was described in Scheme 29 and Scheme 30 [151,152]. The synthesis started with commercially available substituted anthranilic acid **16.1**, which was converted to compound **16.2** by iodination with NIS in DMF. Compound **16.2** was reacted with urea at elevated temperature to obtain quinazolinedione derivative **16.3**. Compound **16.3** was treated with phosphorus oxychloride (POCl_3_) and DIPEA under heating conditions to obtain dichloroquinazoline derivative **16.4** in 50% yield in three steps. Aromatic nucleophilic substitution of more reactive chlorine in compound **16.4** was performed with *tert*-butyl (1*R*,4*R*)-2,5-diazabicyclo [2.2.1]heptane-2-carboxylate in THF in the presence of DIPEA as a base to produce compound **16.5**. It was reacted with 4-hydroxy tetrahydropyran in an S_N_Ar manner using Cs_2_CO_3_ as a base to furnish ether scaffold **16.6**. The reactive fluorine in compound **16.6** was replaced with benzyl alcohol in another S_N_Ar reaction using base-mediated potassium *tert*-butoxide to yield benzyloxy-substituted product **16.7**. The chemo-selective Suzuki coupling reaction of compound **16.7** with cyclopropylboronic acid in the presence of Pd(dppf)Cl_2_ and K_3_PO_4_ produced iodo-substituted product **16.8**. Another Suzuki reaction of compound **16.8** with THP-protected indazole boronic acid pinacol ester **16.9** in the presence of RuPhos Pd G3, RuPhos, and K_3_PO_4_ at 130 °C furnished scaffold **16.10** with moderate yields. Deprotection of the benzyl group on compound **16.10** was treated with Pd/C under a hydrogen atmosphere in methanol to afford compound **16.11**. Final nucleophilic substitution of 1-(chloromethyl)-4-ethynylbenzene with compound **16.11** in the presence of Cs_2_CO_3_ produced the desired alkyne compound **16.12** in reasonable yield.

The final synthesis of ASP-3082 is described in Scheme 30.

The synthesis commenced with commercially available amine **16.13**, which was converted to compound **16.15** through the amidation reaction with acid **16.14** in the presence of HATU and DIPEA in DMF, as mentioned in Scheme 30. Deprotection of -Boc group in compound **16.15** was removed with HCl in 1,4-dioxane, to obtain product **16.16**. The amine **16.16** was reacted with *N*-boc-*L*-valine **16.17** under HATU and DIPEA conditions to give **16.18**. Boc deprotection in compound **16.18** was achieved using HCl in dioxane to generate valyl amide **16.19**. The free amine in compound **16.19** was converted to its corresponding azide utilizing reagent **16.20** in the presence of NEt_3_ in ACN and THF to furnish compound **16.21** at moderate yields. The click reaction of the azide **16.21** and previously synthesized alkyne **16.12** (from Scheme 29) in the presence of sodium ascorbate and copper iodide (CuI) in *tert*-butanol/water at 50 °C yielded triazole scaffold **16.22**. Finally, the deprotection of the Boc and THP groups with TFA in DCM furnished the final compound, **ASP-3082 (16)**, in excellent yield.

### 2.12. PROTACs Targeting Interleukin-1 Receptor-Associated Kinase 4 (IRAK4)

KT-413 is a novel dual-mechanism degrader developed to address the unmet medical need in treating the ABC subtype of diffuse large B-cell lymphoma (DLBCL), particularly in tumors driven by MYD88 mutations. It efficiently degrades IRAK4 and the transcription factors Ikaros and Aiolos by functioning both as a heterobifunctional degrader and a molecular glue. KT-413′s ability to simultaneously target these proteins results in potent inhibition of NF-κB signaling and activation of Type I interferon responses, restoring apoptosis and suppressing tumor growth. Supported by promising preclinical data, KT-413 is now in Phase 1 clinical trials (NCT05233033) for non-Hodgkin’s lymphoma, DLBCL, and MYD88-mutant indications [153].

**Scheme 31:**
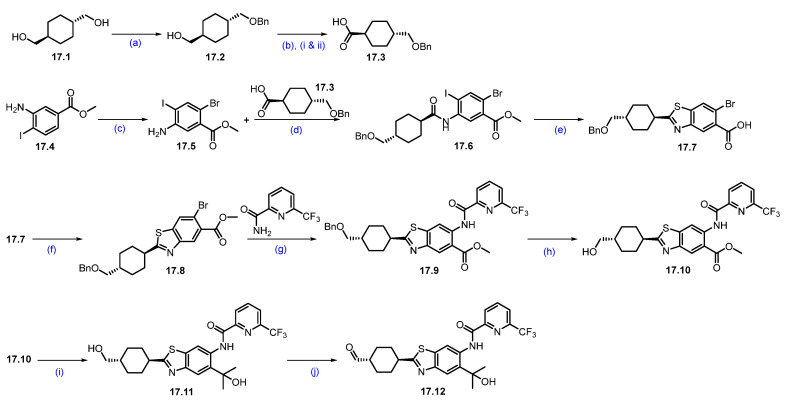
Synthesis of intermediate **17.12** of **KT-413 (17):** (**a**) BnBr, NaH, TBAI, THF, 45%; (**b**) (i) DMP, DCM; (ii) NaH_2_PO_4_, 2-Me-2-butene, NaClO_2_, *^t^*-BuOH, 90%; (**c**) NBS, DMF, 89%; (**d**) T_3_P, pyridine, DCM, 85%; (**e**) CuI, Na_2_S.9H_2_O, TFA, DMF, 95%; (**f**) MeI, K_2_CO_3_, DMF, 69%; (**g**) Pd_2_(dba)_3_, Xantphos, Cs_2_CO_3_, dioxane, 83%; (**h**) BCl_3_, DCM, 90%; (**i**) MeMgBr, THF, 66%; (**j**) DMP, DCM, 85%.

**Scheme 32:**
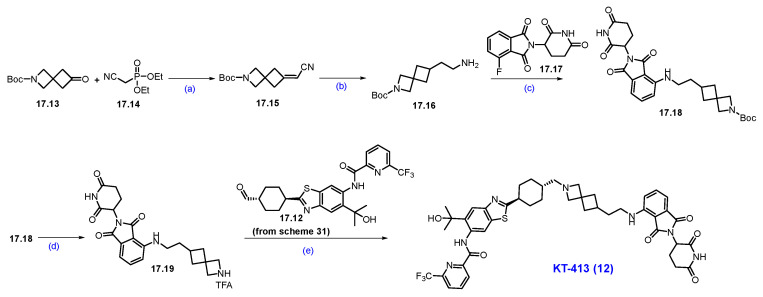
Synthesis of **KT-413 (17):** (**a**) *^t^*BuOK, THF, 66%; (**b**) Raney Ni, H_2_, NH_3_.H_2_O, 67%; (**c**) DIPEA, DMSO, 46%; (**d**) TFA, DCM, 58%; (**e**) TEA, AcOH, NaBH(OAc)_3_, THF, 59%.

Synthesis of **KT-413 (17)**. Recently, Weiss et al. reported the development of KT-413 as a potent degrader that acts as a heterobifunctional degrader and molecular glue. Scheme 31 and Scheme 32 illustrate the synthesis of KT-413, as reported in the literature [154].

The synthesis initiated with commercially available *trans*-1,4-cyclohexanedimethanol **17.1**, which was converted to compound **17.2** by mono-benzyl protection using benzyl bromide and NaH. The primary alcohol of **17.2** was treated with Dess-Martin Periodinane (DMP) to furnish the aldehyde. Further oxidation of the aldehyde functionality under Pinnick oxidation (NaH_2_PO_4_ + NaClO_2_) afforded acid **17.3** in good yield. Next, commercially available substituted aniline **17.4** was subjected to selective bromination with NBS to furnish bromo aniline **17.5**, which was coupled with carboxylic acid **17.3** under T3P conditions, to afford amide **17.6**. Next, the reaction of the amide with sodium sulfide in the presence of CuI, followed by stirring with TFA, led to benzothiazole carboxylic acid intermediate **17.7**. Esterification of acid intermediate **17.7** using MeI, K_2_CO_3_ afforded ester **17.8**. Amination of **17.8** with 6-(trifluoromethyl)picolinamide under Buchwald-Hartwig conditions using Pd_2_(dba)_3_, Xantphos in dioxane furnished amide **17.9**. Deprotection of the benzyl group with BCl_3_ followed by a reaction of methyl magnesium bromide (MeMgBr) afforded tertiary alcohol **17.11**. Finally, oxidation of **17.11** with DMP afforded aldehyde intermediate **17.12** (Scheme 31).

The synthesis of the final degrader started with the commercially available spirocyclic ketone **17.13**, which underwent a base-mediated Wittig homologation with 2-diethoxyphosphorylacetonitrile to produce **17.15**. The resulting alkene **17.15** was subjected to Raney Ni-mediated hydrogenation to render amine **17.16**. Next, intermediate **17.16** was subjected to S_N_Ar reaction with 4-fluoro-thalidomide using diisopropylethylamine in DMSO at 130 °C, producing compound **17.18**. It was treated with TFA to remove the Boc group to afford **17.19** as the TFA salt. Finally, reductive amination of **17.12** with amine **17.19** furnishes the final degrader KT-413 with excellent yield (Scheme 32).

## 3. Outlook and Perspectives

Since 2019, numerous PROTACs have entered clinical trials, primarily for cancer indications, but also for diseases such as Parkinson’s, inflammatory conditions, and chronic pain [154]. The field has advanced rapidly, with three leading candidates now in Phase III clinical trials—the final stage before potential regulatory approval. These late-stage programs target breast cancer, prostate cancer, and leukemia, underscoring the increasing clinical maturity of targeted protein degradation therapies.

Vepdegestrant, which targets estrogen receptors in breast cancer, is the first PROTAC to reach Phase III trials. It is followed by Bavdegalutamide, which aims to degrade the androgen receptor in metastatic prostate cancer, and NX-5948, which targets BTK, a key driver in chronic lymphocytic leukemia.

Despite their promise, PROTACs face several key challenges. These include difficulty targeting membrane-bound proteins, an inability to degrade extracellular proteins, uncertainties about the full biological impact of completely removing a protein, and the risk of off-target degradation.

In summary, targeted protein degradation (TPD) has rapidly emerged as both a robust research platform and a promising therapeutic strategy. As the field evolves, ongoing interdisciplinary collaboration will be crucial to overcoming current hurdles and expanding its clinical reach. Innovations such as the integration of novel E3 ligases, the identification of new binding sites, and the creation of diverse warheads are already setting degraders apart from conventional small molecules. With continued momentum in research and partnership, TPD is poised to drive even more groundbreaking advances in the future.

## Data Availability

No new data were created or analyzed in this study. Data sharing is not applicable to this article.

## Abbreviations

ACN—Acetonitrile; AR—Androgen Receptor; Bcl-xL—B cell lymphoma–extra-large; BCL6—B cell lymphoma 6; BRAF—B-Raf proto-oncogene, serine/threonine kinase; BRD9—Bromodomain-containing protein 9, BTK—Bruton’s tyrosine kinase; CLL—Chronic lymphocytic leukemia CRBN—Cereblon; CRPC—Castration-resistant prostate cancer; DHT—Dihydrotestosterone; DIAD—Diisopropyl Azodicarboxylate; DLBCL—Diffuse large B-cell lymphoma; EDC.HCl—Ethyl-3-(3-dimethylaminopropyl)carbodiimide; DPPA—Diphenylphosphoryl Azide; ER—Estrogen Receptor; GDP/GTP—Guanosine diphosphate/Guanosine triphosphate; HSPs—Heat shock proteins; IAP—Inhibitor of Apoptosis Proteins; IRAK4—Interleukin-1 receptor–associated kinase 4; KRAS—Kirsten rat sarcoma viral oncogene homolog; LRRK2—leucine-rich repeat kinase 2; MCL—Mantle cell lymphoma; MDM2—Murine double minute 2; ORR—objective response rate; PFS—Progression-free survival; Pd_2_(dba)_3_—Tris(dibenzylideneacetone)dipalladium(0); PROTAC—Proteolysis-targeting chimera; RECIST—Response Evaluation Criteria in Solid Tumors; SMARCA2—Switch/sucrose nonfermentable related, matrix associated, actin depended regulator of chromatin, subfamily A, member 2; STAT3—Signal inducer and activator of transcription 3; T3P—Propanephosphonic acid anhydride; TCL—T-cell lymphoma; TPD—Target protein degradation; TPL—Target protein ligand; TPP—Triphenylphosphine; Troc—Trichloroethyl chloroformate; ULL—Ubiquitin ligase ligand; UPS—Ubiquitin-proteasome system; VHL—Von Hippel–Lindau; X-phos—Dicyclohexyl [2′,4′,6′-tris(propan-2-yl)[1,1′-biphenyl]-2-yl]phosphane.
